# Microfluidic Platforms for Ex Vivo and In Vivo Gene Therapy

**DOI:** 10.3390/bios15080504

**Published:** 2025-08-04

**Authors:** Sungjun Kwak, Hyojeong Lee, Dongjun Yu, Tae-Joon Jeon, Sun Min Kim, Hyunil Ryu

**Affiliations:** 1 Department of Biological Sciences and Bioengineering, Inha University, Incheon 22212, Republic of Korea; sungjune.kwak@gmail.com (S.K.); gywjd3527@gmail.com (H.L.); youdongjun7242@gmail.com (D.Y.); 2 Department of Biological Engineering, Inha University, Incheon 22212, Republic of Korea; 3 Biohybrid Systems Research Center (BSRC), Inha University, 100 Inha-ro, Michuhol-gu, Incheon 22212, Republic of Korea; 4 Department of Mechanical Engineering, Inha University, Incheon 22212, Republic of Korea

**Keywords:** nucleic acid-based therapeutics, microfluidics, gene therapy

## Abstract

Recent studies have demonstrated the clinical potential of nucleic acid therapeutics (NATs). However, their efficient and scalable delivery remains a major challenge for both ex vivo and in vivo gene therapy. Microfluidic platforms have emerged as a powerful tool for overcoming these limitations by enabling precise intracellular delivery and consistent therapeutic carrier fabrication. This review examines microfluidic strategies for gene delivery at the cellular level. These strategies include mechanoporation, electroporation, and sonoporation. We also discuss the synthesis of lipid nanoparticles, polymeric particles, and extracellular vesicles for systemic administration. Unlike conventional approaches, which treat ex vivo and in vivo delivery as separate processes, this review focuses on integrated microfluidic systems that unify these functions. For example, genetic materials can be delivered to cells that secrete therapeutic extracellular vesicles (EVs), or engineered cells can be encapsulated within hydrogels for implantation. These strategies exemplify the convergence of gene delivery and carrier engineering. They create a single workflow that bridges cell-level manipulation and tissue-level targeting. By synthesizing recent technological advances, this review establishes integrated microfluidic platforms as being fundamental to the development of next-generation NAT systems that are scalable, programmable, and clinically translatable.

## 1. Introduction

Nucleic acid-based therapeutics (NATs) represent a groundbreaking strategy for treating diseases at their genetic root. They can precisely modulate gene expression or directly correct genetic mutations by using DNA, RNA, or their chemical derivatives. Unlike conventional small-molecule drugs or protein-based therapies, NATs offer the potential to target specific genes at the molecular level, thereby maximizing therapeutic specificity and durability. Major classes of NATs include plasmid DNA (pDNA), messenger RNA (mRNA), small interfering RNA (siRNA), antisense oligonucleotides (ASOs), and the CRISPR-Cas9 (Clustered Regularly Interspaced Short Palindromic Repeats-associated protein 9) system. Each of these modalities exhibits distinct mechanisms of action and therapeutic applications.

Plasmid DNA, typically derived from bacterial circular DNA vectors, can be used to express therapeutic proteins or replace defective genes. Its advantages include ease of production and storage, as well as low immunogenicity [1,2]. mRNA serves as a transient carrier of genetic information from DNA to facilitate protein synthesis within cells. Synthetic mRNA can be delivered into cells to induce the temporary expression of therapeutic proteins, with benefits such as minimal risk of genomic integration, ease of manufacture, and rapid scalability—as exemplified by mRNA-based COVID-19 vaccines [3]. siRNA, composed of 21–23 base-pair double-stranded RNAs, binds precisely to target mRNAs and induces their degradation through the RNA interference (RNAi), effectively silencing gene expression [4,5]. ASOs are 12–25-nucleotide-long single-stranded synthetic oligonucleotides that selectively bind to complementary mRNA sequences, leading to mRNA degradation, translational inhibition, or splicing modulation [6,7,8,9]. The CRISPR-Cas9 system, guided by synthetic single-guide RNAs, facilitates precise genome editing by introducing site-specific double-stranded breaks, enabling gene disruption, correction, or insertion. This technology has been widely applied in treating various genetic disorders and cancers [10]. Collectively, these diverse NATs offer promising alternatives for addressing diseases with underlying genetic and molecular causes that are difficult to treat with conventional modalities. With the recent regulatory approval of multiple NAT-based drugs, their clinical efficacy and safety profiles have been increasingly validated.

Clinically, NATs can be delivered via ex vivo or in vivo gene therapy strategies. In ex vivo gene therapy, a patient’s cells are extracted, genetically modified outside the body, and then re-infused. This approach includes CAR-T cell therapy, TCR-T therapy, and hematopoietic stem cell (HSC) gene therapies, typically employing lentiviral or gamma-retroviral vectors. These modalities have shown remarkable success in treating hematological malignancies and rare genetic disorders, leading to several FDA-approved therapies (Table S1). In contrast, in vivo gene therapy involves the direct delivery of genetic material into the patient’s body, where overcoming biological barriers and achieving efficient delivery to target tissues are key challenges. Lipid nanoparticles (LNPs), polymer-based nanoparticles, and chemically modified molecules such as GalNAc-siRNAs and ASOs serve as prominent delivery systems. Clinically successful examples include mRNA vaccines for COVID-19 (Comirnaty, Spikevax), siRNA therapies for transthyretin-mediated amyloidosis (Onpattro), and ASOs for spinal muscular atrophy (Spinraza), all of which have received FDA approval (Table S2).

These successes highlight the therapeutic potential of NATs in precisely targeting gene expression and correcting defective genes at the cellular and tissue levels. However, the expansion of their clinical applicability remains challenged by several technical limitations. In ex vivo therapy, the use of viral vectors raises concerns such as insertional mutagenesis, immune responses [11,12], and complex manufacturing and regulatory hurdles [13]. While non-viral approaches can address some of these issues, physical methods like electroporation can reduce cell viability [14] and scale-up capacity [15]. Additionally, chemical delivery systems such as LNPs, liposomes, and extracellular vesicles (EVs) often suffer from low reproducibility and variable delivery efficiency across cell types. For in vivo applications, genetic materials face degradation by nucleases in circulation [16,17], poor cellular uptake [18,19], immune recognition [20,21], and off-target distribution [22,23]. These multifaceted challenges underscore the need for delivery technologies that enable precise control over carrier physicochemical properties and biocompatibility.

To address these issues, microfluidic platforms have emerged as promising tools. Microfluidics refers to the manipulation of fluids in micro-scale channels, enabling a broad range of biomedical applications through flexible device design and material selection, such as PDMS and various thermoplastics [24]. This versatility supports integration with high-throughput techniques and scalable parallelization for advanced research and manufacturing. Microfluidic systems can also be integrated with high-throughput techniques such as electric fields, ultrasound, and mechanical compression. Moreover, microfluidics provides a robust platform for producing uniform nanoparticles with tunable size, morphology, and surface characteristics. Lipid nanoparticles (LNPs) synthesized via microfluidic mixing exhibit superior uniformity and reproducibility compared to bulk mixing methods, leading to improved delivery efficiency and tissue specificity in vivo. Although microfluidics has been explored on its own for intracellular delivery and nanoparticle synthesis in numerous reviews [25,26,27,28,29,30,31,32,33,34], an integrative perspective connecting both cellular- and tissue-level delivery strategies remains lacking.

This review aims to bridge this gap by highlighting microfluidic platforms as a versatile solution for both intracellular nucleic acid delivery and carrier fabrication. Specifically, we discuss (1) microfluidic techniques for nucleic acid delivery at the cellular level, including cell deformation, microfluidic electroporation, and sonoporation; (2) strategies for manufacturing and optimizing delivery systems such as LNPs; and (3) emerging integrated delivery approaches, including the production of engineered EVs and cell–hydrogel composites. We further explore how microfluidic platforms—through precise fluid control, high uniformity, reproducibility, and integrative processing capabilities—can enhance the delivery efficiency and targeting specificity of next-generation NATs.

## 2. Microfluidic Platforms for Ex Vivo Gene Therapy

The safe and efficient intracellular delivery of nucleic acids is essential for the success of NATs [30]. In particular, precise control over nucleic acid delivery at the cellular level is critical to maintaining high efficiency while minimizing cellular damage [26]. Microfluidic technologies provide sophisticated physical environments at the micrometer scale. These environments enable diverse physical approaches to transiently disrupt cell membrane integrity. They also facilitate efficient intracellular nucleic acid uptake [25,35]. Following such transient disruption, cells rapidly activate intrinsic membrane repair mechanisms, typically restoring membrane integrity within minutes and minimizing cytotoxicity [36].

**Figure 1:**
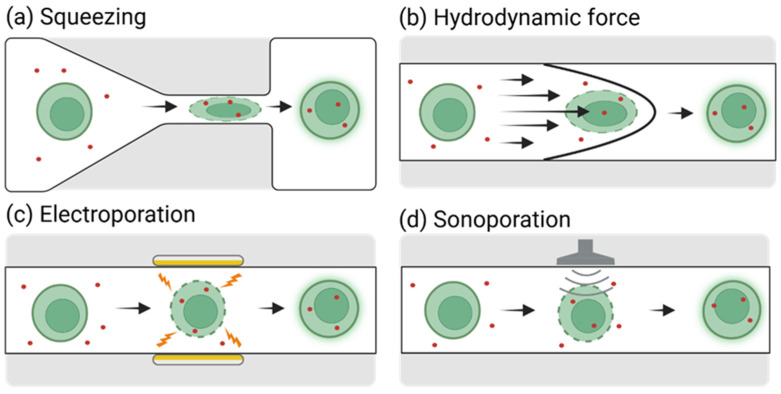
Microfluidic strategies for intracellular nucleic acid delivery. (**a**) Physical squeezing through narrow microchannels for transient membrane permeabilization and efficient intracellular uptake. (**b**) Hydrodynamic deformation-based delivery using extensional and shear stress in structured microchannels. (**c**) Microfluidic electroporation platforms enabling localized electric field exposure. (**d**) Sonoporation-assisted delivery through ultrasound-induced membrane permeabilization. Schematic illustration depicts cells (green) and nucleic acids (red).

Recent advancements employing various approaches, such as cell deformation-based mechanoporation [37,38], microfluidic electroporation [15], and sonoporation [39], have effectively addressed the limitations of conventional techniques related to efficiency and cell viability through microfluidic platforms (Table S3). This section provides a review of the operational principles and recent research achievements in microfluidic-based intracellular nucleic acid delivery technologies that have demonstrated notable technical progress (Figure 1).

### 2.1. Cell Deformation-Based Gene Delivery to Ex Vivo Cells in Microfluidic Platforms

Microfluidic delivery technologies based on cell deformation involve the mechanical induction of transient, reversible pores in the cell membrane to facilitate the intracellular delivery of external materials, such as nucleic acids [35]. These technologies can be broadly categorized into two methods. The first method involves physically compressing cells through microchannels with a width that is narrower than the cell diameter [38]. The second method utilizes hydrodynamic forces within microfluidic channels to deform cells [37].

#### 2.1.1. Mechanical Constriction and Squeezing-Based Gene Delivery

The most direct method for introducing materials into cells through physical deformation involves designing microfluidic channels narrower than the cells, causing mechanical squeezing as cells traverse the channel.

Sharei et al. were the first to demonstrate that physical squeezing of cells through microchannels creates transient membrane pores, thereby enabling the efficient intracellular uptake of external substances. This preliminary study effectively delivered a variety of materials, including carbon nanotubes, proteins, and siRNAs, into HeLa cells, underscoring the versatility and efficacy of the approach [35]. Han et al. (2015) advanced this technology by employing cell squeezing for CRISPR/Cas9 delivery, demonstrating high gene-editing efficiencies in cell types traditionally difficult to transfect, such as lymphoma and embryonic stem cells [40]. Additionally, Li et al. effectively delivered a non-permeable JAK inhibitor into human peripheral blood mononuclear cells (PBMCs) with over 90% efficiency using microfluidic squeezing techniques, outperforming conventional electroporation and passive uptake methods. This result validates the utility of microfluidic squeezing for the intracellular delivery of small-molecule inhibitors with low membrane permeability [41]. Notably, DiTommaso et al. found that cell squeezing induced significantly fewer adverse effects on gene expression profiles and cellular functions compared to electroporation, suggesting that this technique preserves basic cellular functions while achieving efficient delivery [38].

However, early systems based on fixed-width channels exhibited limitations, including clogging due to varied cell size distributions and the need for optimization depending on cell types and cargo properties [35,40]. Subsequent studies addressed these early limitations through the optimization of channel designs and delivery conditions. Saung et al. (2016) demonstrated the selective delivery of nucleic acids and fluorescent molecules to small T-cells (~6.7 µm) and larger cancer cells (~12 µm) by adjusting channel widths, presenting possibilities for selective transfection in samples with broad cell size distributions [42]. Lam et al. identified optimal squeezing conditions in flexible PDMS-based microfluidic channels to efficiently deliver nucleic acids and proteins (3–70 kDa) into human fibroblasts while preserving cell viability [43]. Furthermore, Uvizl et al. introduced a technique to dynamically adjust channel width using external pressure, enabling tailored squeezing conditions based on individual cellular elasticity. This approach effectively delivered both small molecules (4 kDa FITC-dextran) and large complexes (190 kDa Cas9 protein), underscoring the significance of precise condition optimization in squeezing-based delivery systems [44].

**Figure 2:**
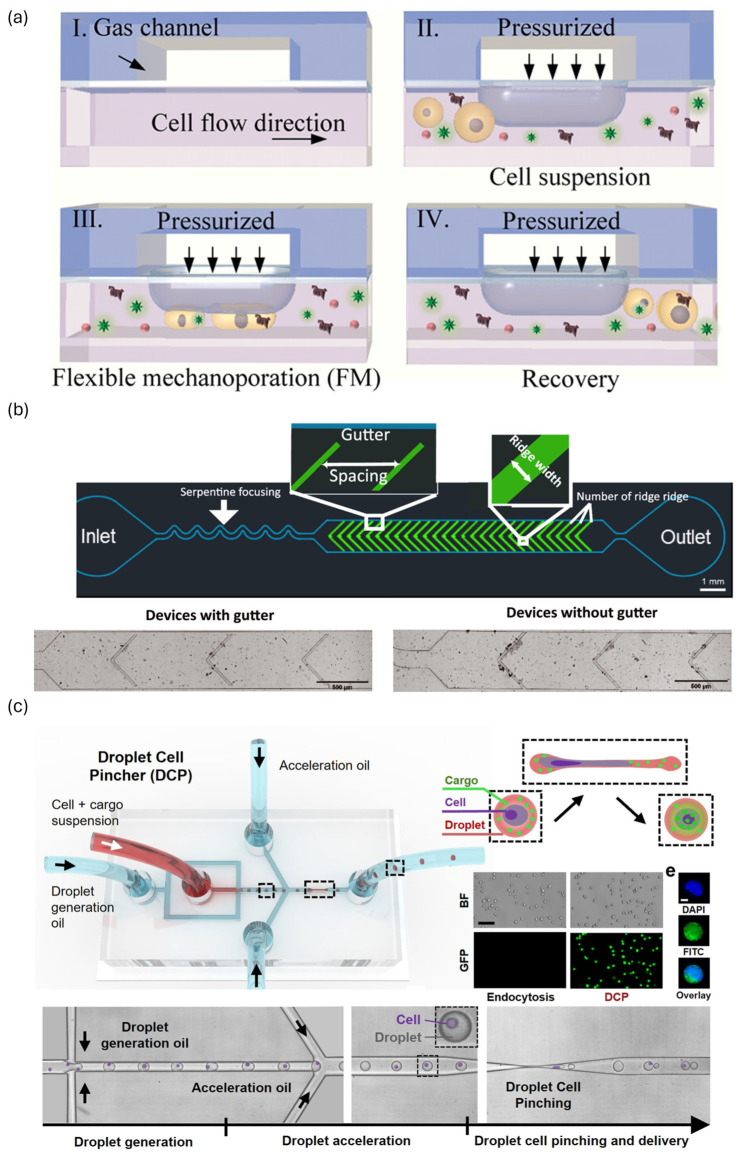
Microfluidic mechanoporation systems address clogging issues. (**a**) A schematic of a flexible mechanoporation chip in which individually actuated pneumatic microvalves dynamically open constrictions, allowing cell clusters to bypass the squeeze zone and prevent clogging during high-throughput delivery. Reprinted and modified from Ref. [46]. (**b**) A schematic of a microfluidic pinching channel featuring gutter sidewalls to passively divert excess cells and maintain continuous single-cell flow. Reprinted and modified from Ref. [47]. (**c**) A schematic of the Droplet Cell Pincher (DCP) system, enabling high-throughput mechanoporation via a single constriction and achieving efficient intranuclear CRISPR delivery. Reprinted and modified from Ref. [48].

Attempts have also been made to address channel clogging in compression-based microfluidic systems. Alhmoud et al. developed a platform utilizing the elastic properties of PDMS channels. In this device, the channel width can be dynamically adjusted in real time by applying pressure to the channel sidewalls via an external linear actuator. When clogging occurs due to the simultaneous entry of multiple cells, this device can temporarily expand the channel to allow the cell cluster to pass, thereby preventing permanent blockage. This dynamic channel system operated reliably with samples containing a wide range of cell sizes and successfully delivered GFP-expressing plasmid DNA into various cell lines with high cell viability. Furthermore, the system enabled the optimization of compression width for each cell type, maximizing delivery efficiency [45]. Building on these advances, Qu et al. introduced a flexible mechanoporation chip system that integrates a three-layer pneumatic microvalve array for high-throughput intracellular delivery (Figure 2a). Unlike the external actuator-based approach of Alhmoud et al., this platform achieves dynamic and adaptive constriction control through chip-integrated pneumatic microvalves, which can be individually actuated to form or release constrictions in real time. When a clog occurs, local pressure increases automatically open the microvalves, allowing aggregated cells to pass and ensuring continuous, clog-free operation without manual intervention. This system supports high-throughput processing with 64 parallel channels, enables the precise optimization of delivery parameters for various cell types, and demonstrates high efficiency and cell viability across a broad range of biomolecules and cell populations, including primary and hard-to-transfect cells. The flexible microvalve design also minimizes mechanical damage compared to rigid channel systems, further enhancing delivery outcomes and operational stability over extended use [46]. Meanwhile, Yu et al. proposed a passive strategy involving structural modification to alleviate clogging. They designed concave gutters on both sides of the microchannel constriction region. When multiple cells enter at once, the excess cells are diverted through lateral outlets (Figure 2b). Consequently, the cells passed through the constriction one by one in a stable manner, ensuring continuous flow. This system was used to deliver CRISPR/Cas9 RNPs into human naïve T cells, achieving over 60% gene editing efficiency while maintaining over 80% cell viability [47]. These approaches, involving either active control or passive structural design, have significantly improved the reliability and throughput of compression-based delivery systems.

Additionally, applying droplet microfluidic techniques to mechanical membrane permeabilization effectively alleviates clogging problems inherent to continuous-flow compression processes. Joo et al. first demonstrated this concept by co-encapsulating cells with external cargoes, such as mRNA and plasmid DNA, in microdroplets, and passing them through a series of micro-constrictions. Within each droplet, recirculating flows induced convective mixing of the surrounding solution, facilitating intracellular delivery. Since the cells were dispersed individually in droplets, channel clogging was largely avoided [49]. However, due to the multi-constriction architecture, droplet stagnation could still occur at high flow rates, resulting in limitations in delivery efficiency for genome editing applications, such as CRISPR. To address these limitations, Kim et al. introduced the Droplet Cell Pincher (DCP) system. The DCP features a single constriction through which droplets pass at high speed (Figure 2c). This design fundamentally resolved the issues of droplet stagnation and channel clogging observed in previous multi-constriction systems. Furthermore, the process enables transient permeabilization of both the plasma membrane and the nuclear envelope, allowing for the direct delivery of CRISPR components to the nucleus. As a result, delivery efficiencies reached 98% for mRNA and 91% for plasmid DNA, along with excellent gene editing performance. Compared to electroporation, the DCP system achieved 6.5-fold higher efficiency in single-gene knockout and 3.8-fold higher efficiency in dual-gene editing and insertion [48].

#### 2.1.2. Hydrodynamic Flow-Based Gene Delivery

Hydrodynamic deformation-based delivery systems leverage extensional [37] and shear stresses [50] induced by microfluidic flow to transiently permeabilize cell membranes without exerting physical pressure on cells through narrow channels. Cells exposed to microfluidic flows experience transient membrane stretching, resulting in the formation of transient nanopores. The formation of these nanopores allows external materials, such as nucleic acids, to diffuse into the cell. This approach is predicated on the premise that it fundamentally prevents channel clogging due to cell size variability and achieves extremely high throughput, processing millions of cells per second due to continuous fluid flow [51,52]. Recent studies have made continuous improvements to delivery performance by optimizing channel geometries and fluid properties to enhance efficiency and cell viability in hydrodynamic deformation-based systems [51,52,53].

Based on these principles of hydrodynamic deformation, the Chung group sequentially refined their intracellular delivery platform. In the initial study by Deng et al., inertial microfluidic flows were utilized to focus cells toward the center of a channel, followed by collision with a wall at a T-junction. This process applied compressive and shear stress to permeabilize the membrane for nanoparticle uptake transiently. This method enabled high-throughput processing of over 1 × 10^6^ cells per minute with high delivery efficiency and approximately 80% cell viability across various cell types [54]. Subsequently, Hur et al. proposed the incorporation of a cavity at the T-junction, facilitating cell stretching through extensional recirculating flows as opposed to direct wall impact. This refinement enabled the effective delivery of large molecules, such as plasmid DNA and mRNA, even into hard-to-transfect primary immune and stem cells. The study achieved up to 98% delivery efficiency with around 90% viability, while maintaining a throughput of ~1 × 10^6^ cells/min [37].

**Figure 3:**
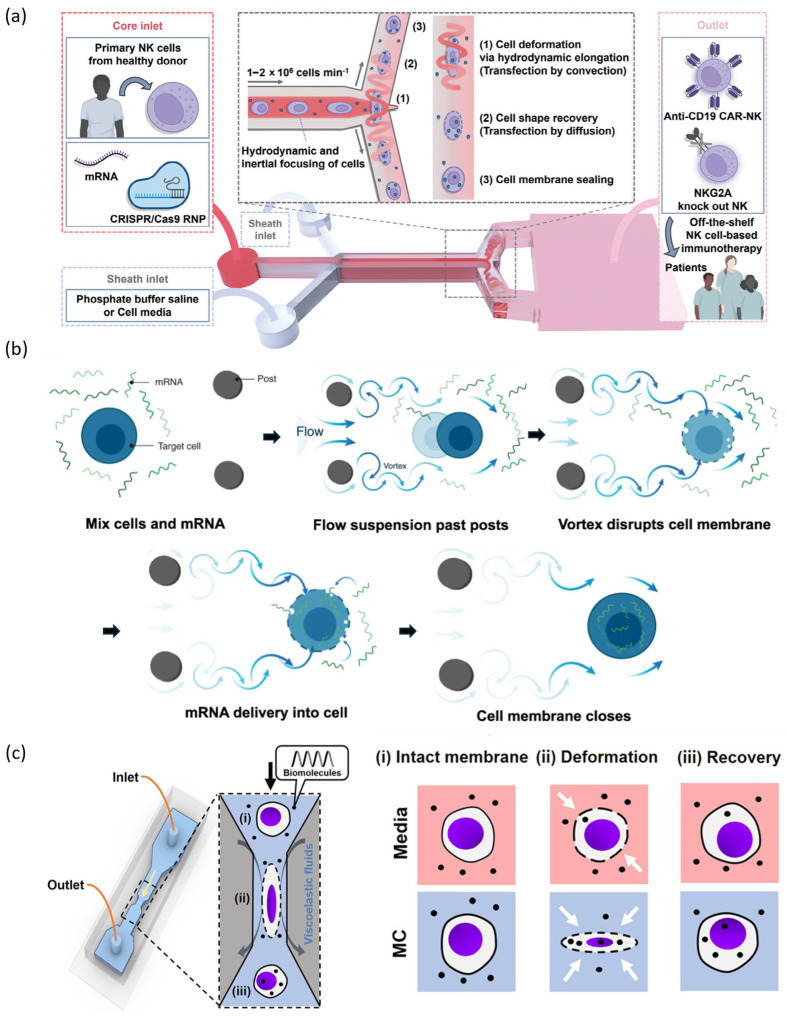
Hydrodynamic deformation-based microfluidic systems. (**a**) A schematic of the Y-hydroporator applying stagnation-induced stretching for efficient CRISPR delivery into NK cells. Reprinted and modified from Ref. [53]. (**b**) A schematic of the microfluidic vortex-shedding (µVS) platform, where vortices generated around microposts transiently permeabilize the membrane to deliver mRNA into human T cells. Reprinted and modified from Ref. [55]. (**c**) A schematic of a viscoelastic flow-based platform combining methylcellulose and single-constriction design to enhance membrane poration and mRNA delivery. Reprinted and modified from Ref. [57].

In a recent study by Kim et al., a Y-shaped microchannel design (Y-hydroporator) was employed to optimize delivery into NK cells. Rapid extensional deformation near the stagnation points at the Y-junction resulted in high delivery and transfection efficiency (Figure 3a), with a throughput of ~2 × 10^6^ cells/min and a long-term cell viability of over 89%. This system was successfully applied to generate CAR-NK cells and genome-edited NK cells, demonstrating clinical potential and scalability [53]. The microfluidic vortex shedding (μVS) method utilizes shear stress induced by vortices forming around microposts, resulting in the transient formation of nanopores in the cell membrane for the purpose of intracellular delivery (Figure 3b). The initial μVS system, as reported in 2019, demonstrated a 63.6% transfection efficiency and 77.3% viability in primary human T cells when delivering EGFP mRNA [55]. A subsequent study by Jarrell et al. refined the design through the use of CFD, incorporating a splitter plate to modulate vortex characteristics and enhancing performance through the addition of brief electric pulses (eμVS). This enhanced system attained gene editing efficiency of up to 38% with Cas9 RNPs in primary human T cells, while preserving >90% viability. It is noteworthy that up to 108 cells could be processed in under 30 s, underscoring its high scalability [56]. In a recent advancement by Sytsma et al., μVS was scaled up for clinical-grade CAR-T manufacturing, achieving a 1.7–2-fold higher yield than conventional electroporation with over 90% viability. CAR-T cells produced by μVS demonstrated comparable functionality in both in vitro and in vivo models, thereby affirming its clinical potential as an alternative manufacturing platform [52]. Recently, the field has focused on viscoelastic fluids, which have demonstrated the capacity to facilitate safe and efficient cell deformation-based nucleic acid delivery [51,57,58]. These fluids exhibit shear-dependent viscosity, thereby enabling the non-contact application of stretching and shear stress to cells in flow. Kwon et al. developed a microfluidic system combining methylcellulose-based viscoelastic fluid and a single-constriction geometry (Figure 3c), achieving up to 97% mRNA delivery efficiency in K562 and Jurkat cells. This approach yielded uniform, transient membrane pores via flow-induced stress, thereby significantly enhancing delivery efficiency and viability in comparison to direct compression-based methods. The platform demonstrated a processing capacity of approximately 3.5 × 10^5^ cells per minute, thereby substantiating its scalability [57]. Sevenler et al. developed a viscoelastic mechanoporation system that utilizes a combination of hyaluronic acid-based viscoelastic fluids and inertio-elastic focusing. This innovative approach facilitates the elongation of cells in a central direction without the need for wall contact, offering a promising avenue for further research in the field. This system facilitated the delivery of mRNA and CRISPR-Cas9 into both Jurkat and primary T cells, with a success rate exceeding 90%, while maintaining viabilities of 85–90% and processing speeds of up to 2.5 × 10^8^ cells per minute. The non-contact deformation process was instrumental in ensuring the stability and high-throughput operation of the system, as evidenced in [51]. Consequently, hydrodynamic deformation-based techniques have emerged as powerful alternatives to conventional compression-based microfluidic systems, offering high viability, excellent throughput, and superior delivery performance.

### 2.2. Electroporation-Based Gene Delivery to Ex Vivo Cells Using Microfluidic Platforms

Electroporation is a technique that introduces nucleic acids and proteins into cells by applying electric pulses that temporarily create nanometer-scale pores in the cell membrane [59]. Traditional bulk electroporation methods have several limitations, including the need for high voltages, substantial cell damage, and poor uniformity [26]. Microfluidic electroporation technologies address these limitations by using precisely engineered microchannels and electrode configurations to control the strength and uniformity of the electric field experienced by each cell locally. This enables high delivery efficiency and cell viability, even at lower voltages [26]. Currently, microfluidic electroporation platforms have evolved into three main categories. The first category is static microfluidic electroporation. In this method, cells are immobilized at specific locations, and electric parameters are finely tuned to optimize delivery efficiency and cell viability [60]. The second category is continuous-flow microfluidic electroporation, which processes cells at a high throughput by flowing them continuously through microchannels [61]. Lastly, selective cell electroporation targets individual cells for electroporation, offering valuable applications in cell therapy and engineering [62].

#### 2.2.1. Static Microfluidic Electroporation Platforms

In static microfluidic electroporation, cells are immobilized on micro- or nanostructures and exposed to electric pulses, allowing for precise control over the intensity and duration of electrical stimulation for each cell [63]. This feature renders the platform optimal for the systematic optimization of conditions that maximize delivery efficiency while minimizing damage [64]. Nevertheless, a significant challenge persists in achieving a balance between efficiency and cell viability, which is contingent upon the voltage and pulse parameters necessary for effective delivery. In response to this challenge, a range of strategies have been formulated. Park et al. conducted a precise measurement of the membrane rupture threshold in microfluidic environments and analyzed the influence of membrane cholesterol content on electroporation performance, thereby aiding in determining optimal settings [60]. Pan et al. conducted a quantitative investigation into Joule heating and electro-osmotic flow effects during localized electroporation through micro- and nanochannels, identifying voltage ranges that minimized damage [64]. Other efforts have focused on localizing electric fields. For example, Ye et al. demonstrated that constriction microchannels could produce highly localized electric fields at the single-cell level. This method maintains high delivery efficiency and viability, even at low voltages [63]. Zhang et al. used a nanostraw system to maintain high viability during repeated electroporation cycles [65]. Patino et al. developed a Live Cell Analysis Device (LCAD) that immobilizes individual cells in microwell arrays and applies localized pulses via nanochannels to reproducibly deliver plasmid DNA and siRNA. They employed deep-learning-based image analysis to correlate morphological characteristics with delivery performance [66]. Much of the existing research has focused on optimizing delivery efficiency through electrical parameters and localized electric field modulation.

**Figure 4:**
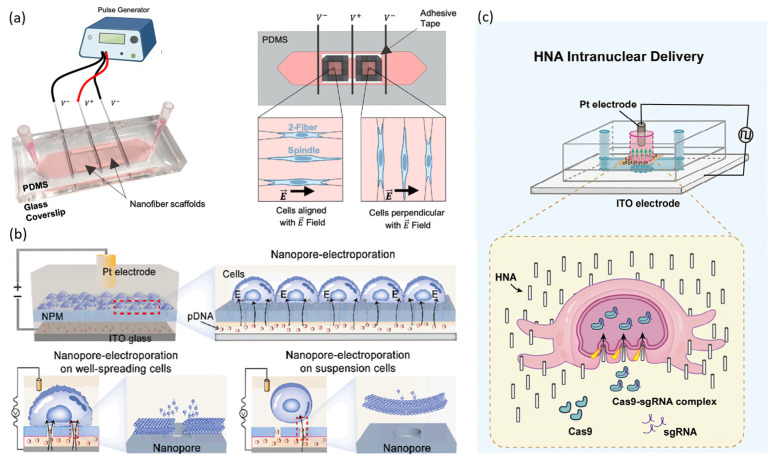
Electroporation strategies based on cell morphology, adhesion, and membrane contact. (**a**) A schematic of nanofiber-aligned cells showing enhanced viability and delivery efficiency when elongated parallel to the electric field. Reprinted and modified from Ref. [67]. (**b**) A schematic illustration of a nanopore electroporation platform emphasizing the importance of cell spreading and adhesion for localized field-mediated delivery. Reprinted and modified from Ref. [68]. (**c**) A schematic of a hollow nanoneedle array-based electroporation system enabling direct intranuclear CRISPR-RNP delivery in dendritic cells. Reprinted and modified from Ref. [69].

However, recent studies have increasingly explored strategies that leverage the morphological and structural characteristics of cells to enhance nucleic acid delivery. For example, Jacobs et al. used nanofiber scaffolds to control cell shape and alignment precisely. They investigated how these factors influenced delivery efficiency and cell viability depending on the cells’ orientation relative to the electric field (Figure 4a). Their study revealed that cells elongated parallel to the electric field exhibited significantly higher post-electroporation viability than cells elongated perpendicular to it. This difference was attributed to variations in local transmembrane potential (TMP) on the cell membrane. Additionally, changes in extracellular buffer conductivity were found to affect cell survival trends [67]. Liu et al. demonstrated that the delivery efficiency of nanopore electroporation (NPE) depends heavily on cellular adhesion and spreading, i.e., how much the cells flatten and adhere to the substrate (Figure 4b). When cells were well spread and firmly attached to the nanopore substrate, the electric field was effectively localized between the cell membrane and the nanopore. This resulted in high delivery efficiency. In contrast, poorly adherent or suspended cells lacked sufficient contact with the substrate, which impaired electric field localization and significantly reduced delivery efficiency. This study underscored the critical role of optimizing adhesion and spreading for the success of nanopore-based nucleic acid delivery platforms [68]. Liu et al. developed a nanostructure-based electroporation system that uses a hollow nanoneedle array (HNA) to address the challenge of poor transfection efficiency in hard-to-transfect cells, such as dendritic cells (DCs). The system used 2-μm-tall hollow nanoneedles to simultaneously contact both the plasma and nuclear membranes (Figure 4c). Localized electrical pulses were then applied to perforate both membranes simultaneously, enabling the direct nuclear delivery of Cas9/sgRNA ribonucleoproteins (RNPs). This approach successfully edited the PD-L1 gene in dendritic cells with significantly higher efficiency than conventional electroporation [69]. Taken together, these static electroporation-based studies suggest that optimizing the balance between delivery efficiency and cell viability requires a multidimensional approach. In addition to electrical parameters, such as membrane rupture threshold and voltage ramp rate [60,63,64], morphological factors must be considered. These factors include TMP modulation through controlled cell alignment [67], enhanced field localization via improved cell adhesion and spreading [68], and the design of nanostructures for simultaneous nuclear and membrane poration [69]. These findings provide important insights for developing next-generation nucleic acid delivery platforms for precision cell therapy and advanced cellular engineering.

#### 2.2.2. Continuous-Flow Microfluidic Electroporation Platforms

Continuous-flow microfluidic electroporation was developed to overcome the low throughput limitation of static systems [15,26]. Traditional batch electroporation processes cells and cargo in discrete volumes, necessitating repeated optimization and extended processing time for large-scale applications [59]. As the scale increases, changes in electrode spacing can alter electric field distributions, necessitating further reoptimization [70]. In contrast, continuous-flow systems allow cells to pass through electric fields as they flow through microchannels [61]. This approach provides uniform exposure to electric fields, simplifies scaling without additional optimization, and improves overall efficiency [13].

**Figure 5:**
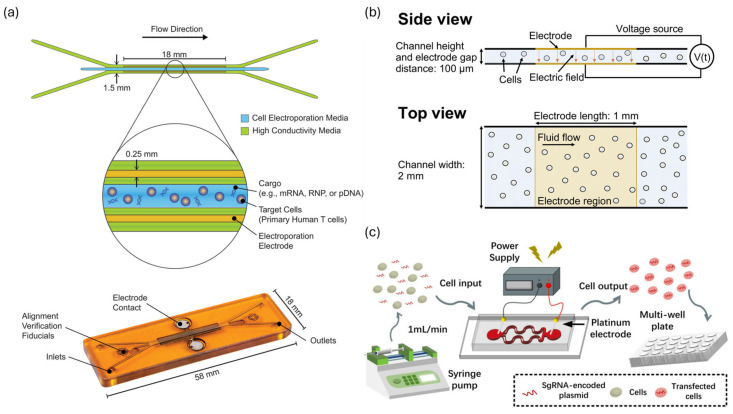
Continuous-flow microfluidic electroporation systems. (**a**) A schematic of a core–sheath flow-based device enabling high-throughput CRISPR/Cas9 delivery into T cells with uniform electric field exposure. Reprinted and modified from Ref. [61]. (**b**) A schematic illustration of a scalable microfluidic electroporation system enabling direct transfer of optimized low-volume pulse conditions to large-volume processing. Reprinted and modified from Ref. [13]. (**c**) A schematic of the LaViE-Chip using hydrodynamic cell rotation for uniform electroporation and efficient gene editing in suspension cells. Reprinted and modified from Ref. [71].

Welch et al. developed a continuous-flow microfluidic device that efficiently delivered CRISPR/Cas9 RNPs and mRNA into primary T cells. The device featured a unique channel design in which the central stream containing cells was flanked by high-conductivity sheath flows in contact with sidewall electrodes (Figure 5a). This configuration ensured a strong, uniform electric field focused on the central stream, protecting cells from electrochemical byproducts. The device maintained consistent exposure conditions and demonstrated exceptional performance, processing ~1.6 × 10^8^ cells per minute with over 93% delivery efficiency and minimal impact on viability [61]. VanderBurgh et al. introduced a system that enables the rapid optimization of pulse conditions for small cell populations and direct scaling up without the need for redesign. By narrowing the electrode gap, the device generated a strong, uniform electric field at low voltages. The structure consistently exposed the sample to the electric field regardless of channel width (Figure 5b), enabling direct translation from small-scale optimization to large-scale processing. Using this platform, plasmid DNA was delivered into Jurkat cells with up to 84% efficiency and 89% viability, with throughput reaching up to 8 × 10^6^ cells per minute [13]. Li et al. developed a system called the LaViE-Chip, which uses hydrodynamic forces to rotate cells and ensure uniform, multidirectional electric field exposure. Multiple parallel microchannels and external electrode placement minimize harmful byproducts (Figure 5c). The curved channel design induced cell rotation, enabling even pore formation across the membrane and improving delivery and viability. The LaViE-Chip system achieved 71% gene editing efficiency and 84% viability using CRISPR/Cas9 RNPs in Jurkat T cells, with a throughput of ~1 × 10^7^ cells per minute [71]. Overall, continuous-flow microfluidic electroporation significantly increases throughput while maintaining high efficiency and cell viability. This technology has great potential as a core platform for the large-scale manufacturing of cell therapies and advanced cell engineering processes.

#### 2.2.3. Target Selective Electroporation Platforms

**Figure 6:**
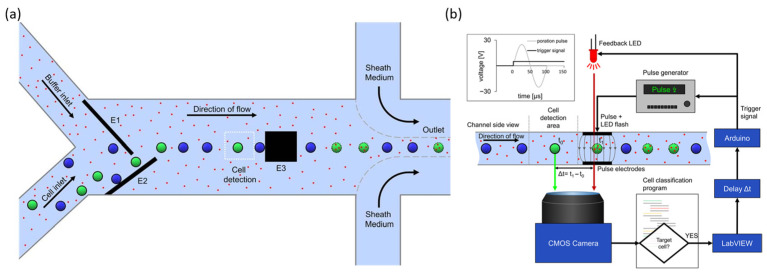
Image-activated selective electroporation. (**a**) A schematic of a microfluidic system enabling real-time image-guided identification and selective electroporation of target cells within mixed populations. (**b**) Workflow and control mechanism of the microfluidic system. Real-time imaging enables cell classification, and a synchronized feedback loop precisely triggers electroporation pulses exclusively to identified target cells. Reprinted and modified from Ref. [62].

Recent advances have made it possible to selectively electroporate specific cells within heterogeneous populations. Pfisterer et al. developed an image-guided, real-time system integrated into a continuous-flow microfluidic chip (Figure 6a). The system uses fluorescence and bright-field microscopy to identify target cells in real time and applies electric pulses only to the selected cells via image-based control (Figure 6b). The authors demonstrated the precise electroporation of target cells based on specific fluorescence markers within mixed populations using this method. The platform achieved over 90% specificity and over 50% delivery efficiency with a processing rate of up to 7200 cells per hour, all while maintaining high cell viability [62]. This approach is valuable for precision cell therapy, single-cell research, and applications requiring precise manipulation.

### 2.3. Sonoporation-Based Gene Delivery Using Microfluidic Platforms

Sonoporation uses ultrasound-induced microbubble oscillation and collapse to temporarily permeabilize cell membranes, allowing the delivery of nucleic acids and other biomolecules [39]. However, conventional sonoporation presents several challenges, including inconsistent bubble size and density [72,73], random bubble arrangement, rapid collapse, and localized shockwave generation, all of which can lead to cell and gene damage. Integrating microfluidics into sonoporation technology provides precise control over ultrasound intensity and focus, microbubble size and distribution, and flow dynamics [74,75]. Specifically, microfluidic environments enable uniform acoustic streaming and consistent cell–microbubble interactions, thereby improving delivery efficiency and cell viability [76].

**Figure 7:**
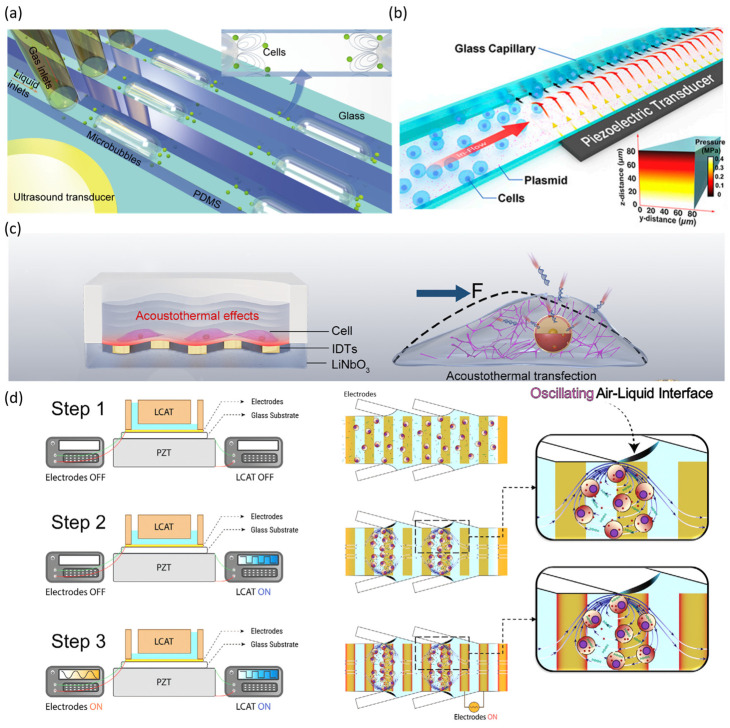
Sonoporation-based microfluidic delivery systems. (**a**) A schematic of a linear array microbubble platform generating uniform microbubbles for stable ultrasound-mediated gene delivery. Reprinted and modified from Ref. [76]. (**b**) A schematic illustration of a microbubble-free acoustofluidic platform using acoustic streaming and radiation force for safe plasmid delivery to primary cells. Reprinted and modified from Ref. [39]. (**c**) A schematic of a SAW-based acoustothermal platform combining thermal and acoustic effects to enhance membrane permeability and transfection efficiency. Reprinted and modified from Ref. [77]. (**d**) A schematic illustration of the AESOP platform integrating acoustic microstreaming and electric fields for high-throughput, dosage-controlled gene delivery. Reprinted and modified from Ref. [78].

Huang et al. developed a linear-array microbubble platform with a vertical T-junction structure and hydrophobic surface treatment. This design generates and stabilizes microbubbles of uniform size and spacing within microchannels (Figure 7a). Traditional sonoporation was limited by irregular bubble behavior; however, this platform enabled stable oscillation and controlled interaction with cells. Acoustic streaming and secondary radiation forces guide cells near the microbubble surface, facilitating uniform exposure. Using 97.5 kHz ultrasound at 30 V for three minutes, green fluorescent protein (GFP) plasmid DNA (~8.2 kb) was delivered into HEK293T cells with 30.7% efficiency and greater than 85% viability [76]. Belling et al. implemented an acoustofluidic platform that achieved ultrasound-mediated sonoporation without microbubbles or contrast agents (Figure 7b). This system relied solely on acoustic radiation forces and streaming within microchannels to apply shear stress to cell membranes and facilitate plasmid DNA delivery. The system demonstrated broad applicability to primary cells, achieving 62% delivery efficiency in Jurkat cells and 15% and 20% efficiency in CD34+ HSPCs and PBMCs, respectively. The system also maintained over 80% viability and processed up to 2 × 10^5^ cells per minute [39].

Liu et al. developed an acoustothermal microfluidic platform that uses surface acoustic waves (SAWs) to increase permeability without the use of microbubbles. The vibrations transmitted through the polydimethylsiloxane (PDMS) layers induced local temperature increases of ~42 °C and acoustic streaming (Figure 7c). These effects increased the permeability of the membrane and nuclear envelope synergistically. The system achieved 89.6% plasmid DNA delivery efficiency into mesenchymal stem cells and primary T cells, maintaining >85% viability. CXCR4-transfected mesenchymal stem cells (MSCs) showed excellent in vivo targeting of ischemic brain regions, supporting the platform’s therapeutic potential [77].

Aghaamoo et al. introduced the AESOP system, which combines acoustic streaming vortices and low-intensity electric fields to achieve synergistic delivery (**z**). Acoustic shear stress formed membrane pores, which electric pulses then exploited for efficient intracellular delivery. The AESOP system enabled high-throughput processing of ~1 × 10^6^ cells/min and achieved 80% GFP plasmid delivery efficiency in HeLa cells while maintaining >80% viability. The system also permitted precise dosage control, demonstrating the potential of hybrid microfluidic stimuli for precise nucleic acid delivery [78]. The continuous advancement of microfluidic sonoporation technologies has significantly improved the precision, efficiency, and safety of nucleic acid delivery.

## 3. Microfluidic Fabricated Nano-Carriers for In Vivo Gene Therapy

**Figure 8:**
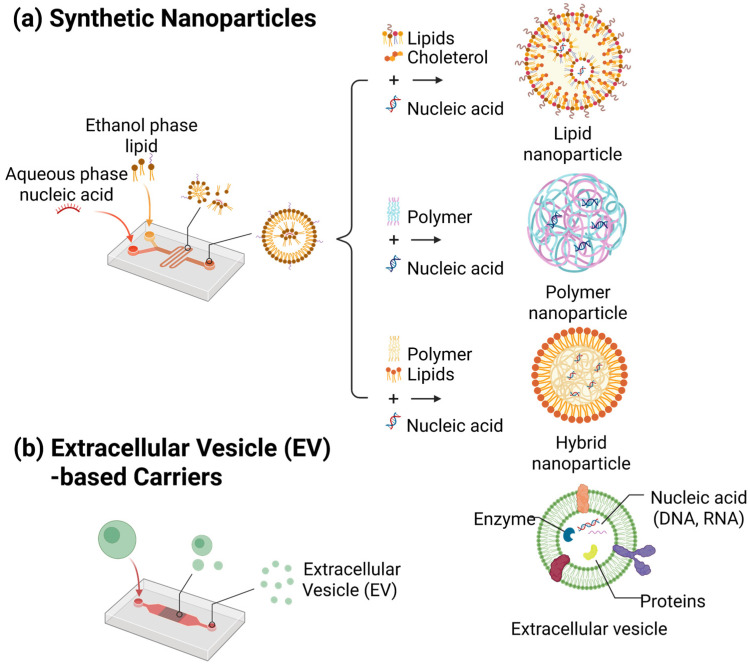
Schematic overview of nucleic acid delivery platforms utilizing (**a**) synthetic nanoparticles and (**b**) EV-based carriers. (**a**) Synthetic nanoparticles: three representative classes of synthetic nanoparticles—lipid, polymer, and hybrid—are generated by mixing an organic phase containing lipids or polymers with an aqueous phase containing nucleic acids within a microfluidic channel. This process enables controlled self-assembly under rapid and precise mixing conditions.

The clinical success of NATs is contingent upon the efficacy and specificity of the carriers in delivering the therapeutic cargo to the intended target tissues or organs [30]. The advent of microfluidic platforms has led to the emergence of highly sophisticated tools for the precise engineering and manufacturing of such carriers, thus ensuring compliance with the aforementioned requirements [31]. Specifically, microfluidic technologies enable precise control over flow dynamics and mixing conditions within micrometer-scale channels during nanoparticle fabrication, thereby allowing consistent regulation of the physicochemical properties of the nanoparticles [34]. Consequently, parameters such as particle size, morphology, and surface characteristics can be more accurately controlled in comparison to conventional methods, thus offering significant advantages for efficient delivery and selective accumulation in target tissues [79]. These physicochemical properties critically influence the cellular uptake mechanisms, as smaller nanoparticles (typically 50–150 nm) are more efficiently internalized via clathrin-mediated or caveolin-mediated endocytosis pathways [80]. Moreover, surface modifications such as PEGylation or ligand conjugation further enhance tissue-specific accumulation by reducing nonspecific clearance and promoting receptor-mediated uptake [81,82]. Microfluidic-based strategies for carrier fabrication can be broadly classified into two categories: synthetic nanoparticles and EV-based carriers. Synthetic nanoparticles, which include LNPs and polymeric nanoparticles, can be meticulously customized in terms of size and surface characteristics through the utilization of microfluidic methodologies. This phenomenon leads to augmented in vivo stability and delivery efficiency [83]. In contrast, EV-based carriers can benefit from microfluidic platforms through significantly increased production yield [84] and improved uniformity, while preserving their natural biocompatibility [85] and intrinsic tissue-targeting capabilities [86]. In recent developments, hybrid systems combining synthetic nanoparticles and EVs via microfluidic technologies have been developed, with the aim of further enhancing carrier performance [33]. In this section, the focus is on the principles of microfluidic-based fabrication of synthetic nanoparticles and EV-based carriers, key processing parameters, strategies for optimizing physicochemical properties, and recent advances aimed at improving targeted delivery efficiency (Figure 8).

### 3.1. Microfluidic Platforms for Nanoparticle Synthesis

Synthetic nanoparticles are among the most widely used carriers for the efficient in vivo delivery of NATs. These carriers offer a multifaceted approach to the protection of nucleic acid drugs from enzymatic degradation, the promotion of selective accumulation in target tissues, and the facilitation of cellular uptake. The delivery performance of synthetic nanoparticles can be further optimized through microfluidic technologies. In this section, we initiate the discussion by introducing the characteristics and current applications of the major types of synthetic nanoparticles. We proceed to a thorough examination of the fundamental principles and critical process parameters that underpin the fabrication of nanoparticles through microfluidic techniques. Subsequent to this, strategies are presented to enhance in vivo biodistribution through the optimization of particle size and uniformity, as well as recent microfluidic approaches for the precise targeting of synthetic nanoparticles.

#### 3.1.1. Types of Synthetic Nanoparticles

The delivery performance and application range of synthetic nanoparticles are contingent upon the materials utilized and their structural characteristics. These particles can be broadly categorized into three distinct classes: LNPs, polymeric nanoparticles, and hybrid nanoparticles. Each type possesses distinct advantages and limitations based on their compositional properties and design strategies.

- Lipid Nanoparticles (LNPs)

LNPs are spherical nanocarriers composed of ionizable cationic lipids, phospholipids, cholesterol, and PEGylated lipids. Typically, these structures are formed through a process of self-assembly, whereby an aqueous solution containing nucleic acids is mixed with a lipid solution in an organic solvent [83]. Due to their lipid bilayer structure, which resembles the cellular membrane, LNPs exhibit high biocompatibility. These particles encapsulate nucleic acids, such as mRNA or siRNA, within their aqueous core, thereby protecting the cargo and enabling efficient delivery [87]. Specifically, ionizable lipids maintain a neutral charge under physiological conditions but acquire a positive charge in the acidic environment of endosomes. This process facilitates endosomal escape through interactions with the endosomal membrane, thereby enhancing intracellular delivery efficiency [88]. The efficacy and safety of LNP-based technologies have been validated by the approval of the first mRNA drug [89] and the success of mRNA vaccines [90]. Consequently, LNPs have become the most prevalent delivery systems for mRNA vaccines and RNA interference (RNAi) therapeutics [83,91]. They are also being utilized for the delivery of microRNAs [92,93], antisense oligonucleotides [94,95], plasmid DNA [96] and mRNA, and guide RNA delivery of CRISPR-Cas9 systems [97,98].

- Polymeric Nanoparticles

Polymeric nanoparticles are nanocarriers composed of synthetic or natural polymers. These systems have been shown to encapsulate nucleic acids within a biodegradable polymer matrix or form polyplexes through electrostatic interactions between cationic polymers and anionic nucleic acids [99,100]. The physicochemical properties of these carriers, including particle size, surface charge, and degradation rate, can be meticulously adjusted by manipulating the chemical structure and molecular weight of the polymers. This allows for the development of customized designs, such as controlled release profiles and prolonged circulation times. For instance, biodegradable polymers such as PLGA offer tunable degradation and release durations ranging from several days to weeks, depending on their composition [101]. It has been demonstrated that certain cationic polymers, including polyethyleneimine (PEI) [102] and chitosan [103], possess the capacity to disrupt endosomal membranes under acidic conditions. This process has been shown to promote endosomal escape and enhance delivery efficiency [104]. Polymeric nanoparticles, in their capacity as non-viral delivery vectors, have been demonstrated to exhibit low immunogenicity and are amenable to large-scale production. These vectors are capable of accommodating a wide range of nucleic acids, including small oligonucleotides and multi-kilobase plasmids, rendering them suitable for various applications, such as plasmid DNA vaccines [105], siRNA treatments [106], and more recently mRNA vaccines [107,108] and CRISPR-Cas9 RNP delivery [109].

- Hybrid nanoparticles

Hybrid nanoparticles are engineered by integrating two or more distinct materials into a single nanostructure, thereby conferring enhanced properties beyond those of particles composed of a single material [110]. A notable example is the lipid–polymer hybrid nanoparticle, which generally comprises a polymeric core encased within a phospholipid bilayer shell. This configuration combines the biomimetic properties of LNPs—such as membrane-like biocompatibility—with the mechanical stability of polymeric nanoparticles. These hybrid structures facilitate the encapsulation and stable delivery of nucleic acids, while concurrently promoting cellular uptake through the lipid exterior [111,112]. The integration of the distinct advantages inherent in each component enables hybrid carriers to surpass single-material systems in terms of delivery efficiency. Moreover, the precise calibration of compositional ratios and surface characteristics enables the customization of pharmacokinetic behavior, cell-specific targeting, and controlled intracellular release [113]. The incorporation of inorganic nanomaterials, including gold, iron oxide, and silica, into lipid/polymer matrices serves to expand the functional repertoire of these carriers. This expansion is achieved by enabling imaging, magnetic manipulation, and other auxiliary functions. Consequently, hybrid nanoparticles are positioned as valuable platforms for theranostic applications [114,115]. However, the utilization of pure inorganic nanoparticles as nucleic acid carriers remains constrained, primarily due to concerns regarding biocompatibility [111,116]. These particles are predominantly employed as auxiliary components within hybrid systems.

#### 3.1.2. Key Parameters for the Fabrication of Nanoparticles

The fundamental principle of nanoparticle fabrication using microfluidics involves the mixing of an organic phase—which contains lipids or polymers—with an aqueous phase that includes the nucleic acid payload. This process occurs within microchannels. The efficacy of mixing can be achieved without the employment of highly complex techniques by merging the two solutions at the T-junction [117,118] or Y-shaped microchannel intersection [119]. Furthermore, the incorporation of zigzag-shaped obstacles or three-dimensional structures within the channels has been shown to induce chaotic advection, thereby significantly enhancing mixing efficiency [120,121]. During the mixing process, rapid dilution of the organic solvent by the surrounding aqueous phase leads to nano-precipitation of lipids or polymers in an insoluble form [122], or promotes the self-assembly of these components into nucleic acid nanoparticles, typically measuring tens of nanometers in diameter. In microfluidic systems, two key parameters—flow rate ratio (FRR) and total flow rate (TFR)—play crucial roles in controlling particle formation and determining their physicochemical properties. The FRR, defined as the ratio between the aqueous and organic phases, significantly affects the nucleation and growth of nanoparticles by modulating local solvent composition and supersaturation levels during mixing. For example, a higher aqueous-to-organic FRR results in the rapid dilution of the organic solvent, causing lipids or polymers to quickly reach supersaturation and undergo multiple nucleation events. This typically yields smaller particles [118]. In contrast, increasing the proportion of the organic phase enhances mixing efficiency and tends to produce larger particles. In the case of lipid–polymer hybrid nanoparticles, Santhanes et al. demonstrated that both particle size and surface charge varied significantly with changes in the FRR. Specifically, at a low FRR (e.g., 3:1), the resulting particles tended to be larger and exhibit a higher positive surface charge. In contrast, a higher FRR (e.g., 5:1) leads to smaller particle sizes and reduced surface charge, which can help lower cytotoxicity and improve nucleic acid delivery efficiency [123]. In addition to FRR, TFR plays a key role in determining the residence time and shear force experienced by fluids within microfluidic channels. Increasing the TFR shortens the time the fluids spend in the mixing region, while simultaneously enhancing shear-driven mixing. This promotes rapid nucleation and limits particle growth, thereby resulting in the formation of smaller and more monodisperse nanoparticles. Conversely, lower TFRs can lead to slower mixing and longer particle growth periods, often yielding larger particles. In a study by Hong et al., the TFR was varied from 1.2 mL/min to 68 mL/min while maintaining a constant ratio between the lipid and aqueous phases during LNP synthesis. The results clearly demonstrated that increasing the flow rate led to a reduction in particle size and an improvement in size uniformity [124]. Moreover, it has been reported that even with identical LNP formulations, the optimal flow conditions (FRR and TFR) can vary depending on the type of nucleic acid cargo. For instance, in a study comparing LNPs encapsulating GFP mRNA with those encapsulating CRISPR-Cas9 guide RNA, Palanki et al. found that the optimal mixing conditions differed between the two systems. They also observed that the flow rate range yielding the highest delivery efficiency varied accordingly [125]. Recently, not only have FRR and TFR been validated as critical experimental parameters, but their optimization and analysis are also being pursued through advanced computational approaches. For example, CFD simulations have been employed to examine flow patterns and mixing efficiency under different FRR and TFR conditions in microfluidic LNP production [126,127], and machine learning models (e.g., XGBoost, ANN) have been applied to optimize LNP formulation by exploring various composition ratios, flow rates, and nucleic acid types [128,129]. This demonstrates that microfluidic platforms enable precise process optimization tailored to the physicochemical properties of materials, reflecting the high level of control achievable through microfluidic manufacturing. Based on this principle, a variety of nucleic acid delivery systems—such as LNPs, polymeric particles, and hybrid particles—can be readily produced without the need for additional specialized equipment. Owing to the improved particle uniformity and reproducibility, microfluidic technology has found broad applications, ranging from research reagents to clinically relevant nano pharmaceuticals [124,125,127,130].

#### 3.1.3. Optimizing Particle Size and Uniformity Control of Nanoparticles

In the microfluidic-based synthesis of LNPs, the dimensions and uniformity of the particles are critical factors that influence in vivo delivery efficiency and tissue-specific distribution. Peng et al. employed microfluidic mixing techniques to produce DOTAP-based LNPs by leveraging the rapid mixing dynamics within a precisely engineered microfluidic chip. Specifically, the system employed a T-junction configuration, in which lipid and plasmid DNA solutions intersected perpendicularly, thereby enabling rapid and homogeneous mixing. Consequently, they effectively produced uniform nanoparticles with an average size of approximately 80 nm and minimal aggregation. The LNPs produced via this method exhibited an encapsulation efficiency that was significantly higher than that of those produced via the conventional lipid film method. Furthermore, the study demonstrated enhanced accumulation and nucleic acid delivery efficiency specifically to the lungs and spleen. It is important to note that the delivery efficiency to these target organs was enhanced by approximately two to threefold compared to the lipid film approach [131]. Similarly, Petersen et al. demonstrated the benefits of using microfluidic techniques for producing LNPs. Using the NanoAssemblr™ Benchtop microfluidic mixing system, they precisely combined various ionizable lipid components to produce particles with a uniform size ranging from 80 to 120 nm. Unlike manually mixed particles, which were larger (up to 240 nm) and had broader size distributions, the microfluidic-produced LNPs were more uniform with lower inter-particle variation. Consequently, they achieved a fourfold increase in liver-specific delivery efficiency. Furthermore, the nonspecific distribution observed with manual mixing was substantially reduced with the microfluidic approach. These results show that the unique architecture and precise flow control of microfluidic platforms directly influence LNP size and uniformity. This enables efficient in vivo delivery and enhanced tissue-specific targeting [79].

#### 3.1.4. Tissue-Specific Targeting Enabled by Microfluidic Platform

**Figure 9:**
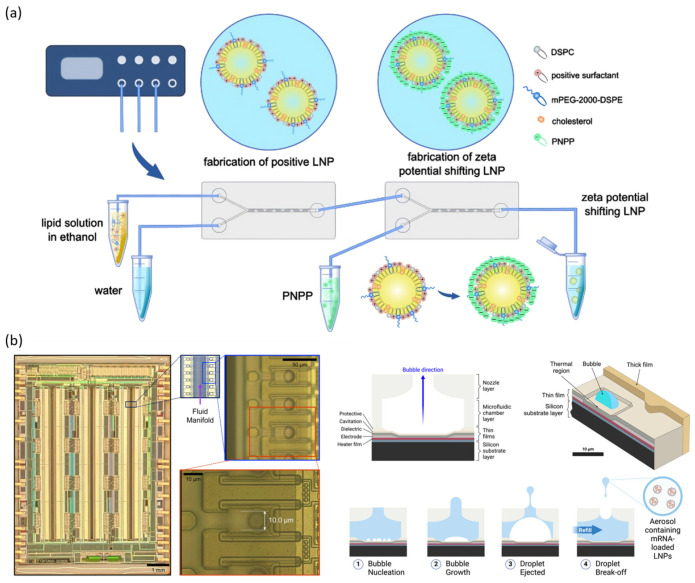
Microfluidic strategies for nanoparticle surface modification and aerosol delivery. (**a**) A schematic of a two-step microfluidic coating process for dynamic surface charge conversion of LNPs using cationic lipid formation followed by anionic PNPP coating. Reprinted and modified from Ref. [81]. (**b**) A schematic illustration of a microfluidic aerosolization platform (MAP) generating shear-free aerosol droplets for the efficient pulmonary delivery of mRNA-loaded LNPs. Reprinted and modified from Ref. [134].

Commercially available microfluidic systems such as NanoAssemblr have been widely utilized to reproducibly fabricate LNPs due to their robust fluidic control and rapid mixing capabilities [132]. While such platforms have achieved established commercial adoption for the consistent production of small molecules, ongoing research continues to expand their functionalities toward improved tissue-specific delivery and enhanced therapeutic efficacy. Current studies emphasize functional advancements, particularly by uniformly modifying nanoparticle surfaces to enhance targeting specificity and therapeutic outcomes. Specifically, the precise fluid control, rapid mixing, and accurate regulation of flow rates intrinsic to microfluidic devices enable the design of innovative particles with high targeting specificity toward desired tissues. Naidu et al. established a comprehensive LNP library using a NanoAssemblr incorporating various ionizable lipids. The uniform mixing dynamics and precise control inherent to microfluidic technology enabled consistent and reproducible formulation, even when introducing subtle structural variations in lipid components. As a result, Lipid 16 demonstrated remarkably high specificity for CD11bhi macrophages without the need for additional targeting ligands, while Lipid 23 exhibited superior delivery efficiency to liver tissue [133]. These findings highlight the potential of precise microfluidic-based nanoparticle fabrication combined with library screening approaches for the development of cell-specific targeting strategies. Zöller et al. proposed a unique strategy involving two microfluidic chips connected in series to sequentially modulate the surface characteristics of nanoparticles. In the first chip, uniform cationic nanoparticles were synthesized using DOTAP-containing lipid formulations. In the second chip, these nanoparticles were coated with a negatively charged PNPP phosphate ester surfactant (Figure 9a). The resulting particles, bearing a negatively charged surface, minimized electrostatic interactions with the mucus layer and effectively penetrated the mucosal barrier. Upon reaching epithelial surfaces, the enzymatic degradation of PNPP led to a charge reversal, restoring the nanoparticle surface to a cationic state, which significantly enhanced cellular uptake [81]. This sequential microfluidic process enabled dynamic surface modification, resulting in markedly improved in vivo delivery performance of the nanoparticles.

Kim et al. developed a microfluidic aerosolization platform (MAP) to efficiently deliver mRNA to the lungs. MAP was designed to generate precisely controlled aerosols through a microscale nozzle array embedded within a microfluidic chip (Figure 9b) to address the structural damage to nanoparticles and mRNA leakage caused by the high shear stress associated with conventional vibrating mesh nebulizers. This design enables low-shear aerosolization, preserving the physicochemical integrity of the nanoparticles and the encapsulation efficiency of the mRNA. Consequently, the nanoparticles aerosolized via MAP exhibited high delivery efficiency to the lungs. In vivo experiments further confirmed selective pulmonary targeting and successful mRNA expression following delivery [134]. Together, these findings underscore how the precise fluid manipulation and specialized designs of microfluidic platforms allow for fine-tuning of the nanoparticle surface characteristics and internal composition, thereby enhancing targeted delivery performance to specific tissues and cell types.

### 3.2. Extracellular Vesicles (EVs) and EV–Nanoparticle Hybridization

Extracellular vesicles (EVs), which are naturally derived nanoparticles secreted by virtually all cell types, have recently emerged as promising carriers for the efficient in vivo delivery of NATs [30]. Exosomes, which range from 30 to 150 nm in diameter, are one type of EV generated through the endosomal multivesicular body (MVB) pathway [135]. Exosomes possess a lipid bilayer structure composed of membrane proteins, such as tetraspanins, and lipids. They also encapsulate abundant proteins and nucleic acids originating from their parent cells [135]. Due to their inherent biological properties, EVs have several advantages over synthetic nanoparticles. These advantages include lower toxicity, improved biocompatibility [85], and reduced clearance by complement activation or macrophage uptake [136]. Ultimately, this results in a prolonged circulation half-life. Additionally, certain EVs exhibit natural homotypic targeting capabilities via parent cell-specific surface markers, enabling selective uptake by the same cell type or preferential accumulation in specific tissues [137]. This feature enables effective delivery across biological barriers, such as the blood–brain barrier (BBB) [138], and into complex environments, such as the tumor microenvironment, thereby enhancing targeting specificity [86]. Despite their biological advantages, naturally secreted EVs have several technical limitations, including low production yields and poor size and composition uniformity [84]. These challenges have hindered their clinical translation and large-scale manufacturing, emphasizing the need for improved production technologies. Despite the fact that a significant portion of the preliminary microfluidic research on EVs has focused on detection and isolation [139,140,141], there is a mounting trend towards leveraging microfluidic platforms to enhance production yield and nucleic acid delivery efficiency. Recently, microfluidic platforms have emerged as powerful tools that can dramatically enhance EV yield [84] and enable precise control over particle uniformity and nucleic acid loading efficiency [142]. Concurrently, there has been growing interest in hybrid delivery systems that combine EVs with synthetic nanoparticles as an innovative strategy that leverages the biocompatibility of natural carriers and the structural tunability of synthetic systems. This section highlights recent advances in microfluidic technologies that aim to (i) significantly improve the scalability and uniformity of EV production, (ii) optimize nucleic acid loading efficiency into EVs, and (iii) enhance delivery functionality through EV–synthetic nanoparticle hybridization.

#### 3.2.1. EV Production and Purification via Microfluidic Platforms

**Figure 10:**
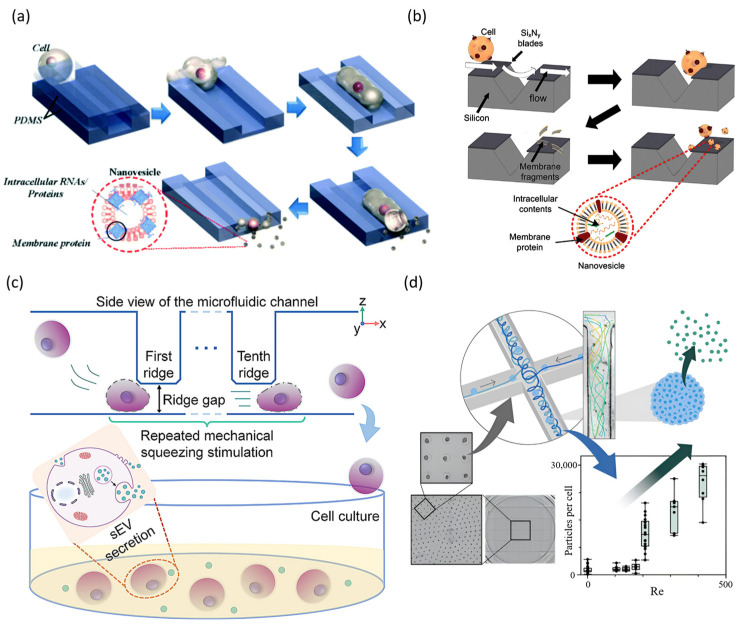
Microfluidic production platforms for cell-derived EVs. (**a**) A schematic of a hydrophilic microchannel-based device generating nanovesicles via direct cell extrusion for RNA/protein-rich vesicle production. Reprinted and modified from Ref. [143]. (**b**) A schematic illustration of nanoblade-integrated microfluidic channels for slicing cell membranes to produce bioactive nanovesicles. Reprinted and modified from Ref. [144]. (**c**) A schematic of a ridge-array microchannel system applying cyclic deformation to enhance small EV secretion from stem cells. Reprinted and modified from Ref. [145]. (**d**) A schematic illustration of a vortex-induced millifluidic device promoting EV release from stem cell spheroids with minimal cell damage. Reprinted and modified from Ref. [84].

One of the major technical challenges in developing EV-based therapeutic delivery systems is the low productivity and heterogeneity of the vesicles obtained through conventional natural secretion methods. Microfluidic platforms have recently emerged as a powerful alternative, capable of significantly improving the yield and quality of EVs. Microfluidic technology provides precise control over the physical microenvironment that cells experience, allowing for the precise regulation of shear stress, mechanical deformation, and hydrodynamic stimulation. Microfluidic systems leverage this control to facilitate the scalable, uniform production of EVs with enhanced consistency. Jo et al. introduced a direct production method for cell-derived nanovesicles using microfluidic technology. In their initial study, cells were extruded through narrow, hydrophilic microchannels within a microfluidic chip to generate uniform nanovesicles, which were approximately 100–120 nm in diameter (Figure 10a). These nanovesicles naturally encapsulated mRNA and proteins from their parent cells. They exhibited high bioactivity and achieved a production yield over 100 times higher than naturally secreted Evs [143]. In a subsequent study, the same group developed a modified microfluidic chip equipped with nanoscale blades (“nanoblades”) that physically sliced the membranes of flowing cells (Figure 10b). The resulting membrane fragments spontaneously reassembled into spherical nanovesicles ranging from 100 to 300 nm in diameter. These nanovesicles maintained uniformity and high bioactivity while retaining a rich content of parent cell-derived mRNA and proteins [144]. Together, these studies demonstrate that microfluidic approaches can enable the efficient, scalable production of functional cell-derived nanovesicles. Hao et al. proposed a microfluidic device in which cells pass repeatedly through constriction ridges, undergoing cyclic mechanical stretching (Figure 10c) [145]. This method increased the yield of small EVs derived from stem cells by approximately fourfold compared to conventional culture-based methods. Notably, this enhancement was achieved without causing significant cell damage, and the biological activity of the EVs was preserved.

Thouvenot et al. proposed an innovative method for producing EVs in high yields from stem cell spheroids. This method leverages millimeter-scale vortex flows generated within a microfluidic platform (Figure 10d) [84]. This microfluidic, chip-based system precisely controls the shear stress within the spheroids, stimulating EV secretion while minimizing direct mechanical damage or deformation to the cells. As a result, approximately 30,000 EVs were produced per stem cell, representing a yield increase of over 100-fold compared to conventional, two-dimensional, culture-based secretion. The EVs generated by this approach retained abundant bioactive molecules derived from the parent cells and exhibited excellent proangiogenic and tissue-regenerative properties. This study underscores the potential of microfluidic systems to precisely regulate hydrodynamic stimulation for large-scale EV production while preserving high bioactivity and minimizing cellular damage. Together, these microfluidic technologies demonstrate their ability to dramatically increase EV yield while improving uniformity and reproducibility. Thus, microfluidic EV production platforms are expected to be indispensable for the scalable manufacturing and commercialization of therapeutic EVs.

#### 3.2.2. Gene Encapsulation in EVs via Microfluidic Platforms

The efficient loading of nucleic acids into EVs is critical for their successful application as delivery vehicles. However, conventional loading techniques, such as electroporation [146] and sonication [147], often have low efficiency and may damage the structure of EVs or cause them to aggregate, thereby limiting their practical utility. Microfluidic platforms have been explored as a promising alternative to overcome these limitations.

**Figure 11:**
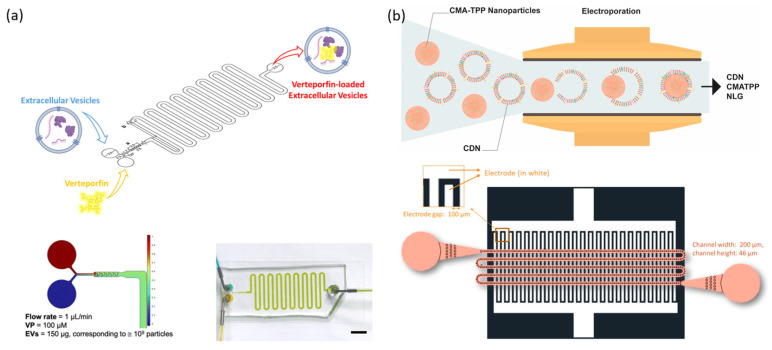
Microfluidic platforms for drug and RNA loading into EVs. (**a**) A schematic of a microfluidic device enabling high-efficiency loading of verteporfin into EVs while maintaining vesicle integrity. Reprinted and modified from Ref. [148]. (**b**) A schematic illustration of a microfluidic electroporation system for coating nanoparticles with EV membranes and co-loading RNA. Reprinted and modified from Ref. [142].

For example, Piunti et al. showed that using a microfluidic device greatly increased the encapsulation efficiency of the poorly soluble drug verteporfin (VP) in EVs, achieving a loading efficiency of about 37.9% compared to ~3.5% using standard passive mixing methods (Figure 11a) [148], This remarkable improvement was attributed to the platform’s ability to provide efficient mixing and precise control over reaction time, thereby preserving the structural integrity and functional properties of EVs during the loading process. Ongoing research continues to explore microfluidic strategies for EV-based nucleic acid delivery. Liew et al. successfully employed a microfluidic electroporation system to simultaneously coat nanoparticles with cell-derived nanovesicle (CDN) membranes and load RNA into their interior (Figure 11b). This system enabled precise control of electrical stimulation, allowing RNA to be stably encapsulated while maintaining the functionality of surface membrane proteins. Compared to conventional methods, the platform also offered improved process automation and scalability [142].

Itakura et al. successfully used a microfluidic device to load small interfering RNA (siRNA) into grapefruit-derived extracellular vesicles (GEVs). Although plant-derived EVs are recognized for their high biocompatibility and cost-effectiveness, they traditionally suffer from low nucleic acid loading efficiency. The researchers achieved uniform and efficient siRNA incorporation into GEVs by precisely controlling the flow rate and pressure within the microfluidic device, improving the loading efficiency to approximately 11%, compared to the lower efficiencies obtained with conventional methods. The resulting siRNA-GEVs effectively suppressed the expression of target genes in HaCaT cells, demonstrating the functional delivery capability and practical potential of microfluidic-assisted nucleic acid loading into Evs [149]. Together, these studies clearly show that microfluidic technology meaningfully enhances the loading and delivery efficiency of nucleic acids in EV-based systems. This underscores the growing importance of microfluidic technology in developing NATs and translating EV-based therapies to the clinic.

#### 3.2.3. Functional Enhancement via EV–Nanoparticle Hybridization

Due to their naturally derived characteristics, EV-based delivery systems face inherent limitations in controlling size, composition, and structural uniformity, making it challenging to engineer them precisely for specific therapeutic purposes. Recent strategies have focused on creating hybrid systems by combining EVs with synthetic nanoparticles to overcome these limitations. This approach enhances the targeting efficiency and biocompatibility of the carriers. Microfluidic technologies, such as microfluidic mixing, electroporation, and sonication, enable the precise fabrication of hybrid nanoparticles. This method offers significant advantages over conventional bulk methods in terms of uniformity and production efficiency.

**Figure 12:**
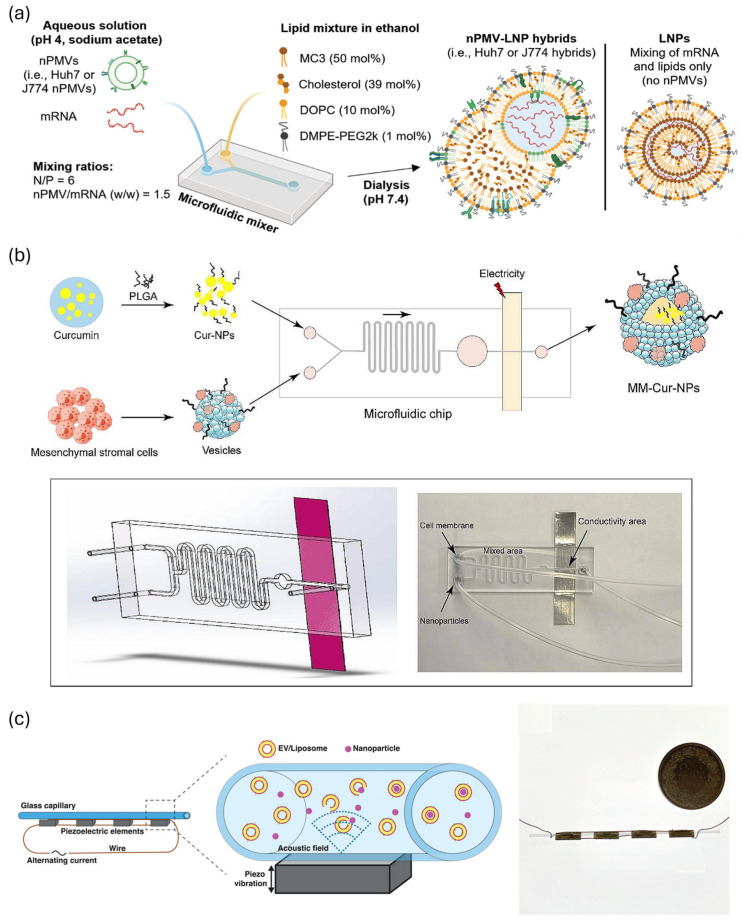
Microfluidic fabrication of nanoparticle–vesicle hybrids. (**a**) A schematic of a staggered herringbone mixer-based microfluidic system for assembling nPMV–LNP hybrids with high uniformity and enhanced intracellular delivery. Reprinted and modified from Ref. [33]. (**b**) A schematic illustration of a microfluidic electroporation platform enabling fusion of neuron-like stem cell membranes with PLGA–curcumin nanoparticles for neuroprotective therapy. Reprinted and modified from Ref. [150]. (**c**) A schematic of a microfluidic μSonicator enabling efficient EV/liposome coating on PMMA nanoparticles with high uniformity and coating efficiency. Reprinted and modified from Ref. [151].

For example, Alter et al. developed a hybrid nanoparticle (nPMV-LNP) by mixing cell-derived nanoplasma membrane vesicles (nPMVs) and LNPs with a staggered herringbone microfluidic platform (Figure 12a). The resulting hybrid particles exhibited a uniform size distribution of approximately 120 nm and a polydispersity index (PDI) below 0.2. Compared to conventional LNPs, these hybrid nanoparticles demonstrated superior intracellular drug delivery efficiency and enhanced in vivo mRNA expression. This highlights the crucial role of microfluidic mixing in improving the structural consistency and biological functionality of nanoparticles [33]. Using microfluidic electroporation, Lei et al. developed curcumin-loaded poly (lactic-co-glycolic acid) (PLGA) hybrid nanoparticles coated with neuron-like cell membranes derived from mesenchymal stem cells (MSCs), termed MM-Cur-NPs (Figure 12b). This approach enabled the precise fusion of the cell membranes with the nanoparticles, creating hybrid structures that can exert multiple therapeutic effects simultaneously in a Parkinson’s disease model. These effects include dopaminergic neuronal protection, anti-inflammatory activity, and restoration of mitochondrial function [150]. In another study using microfluidic sonication, Pareja-Tello et al. combined microfluidic mixing and sonication to create hybrid nanoparticles made of mesenchymal stem cell (MSC)-derived EVs and mRNA-loaded LNPs, known as MSC-EV hybrids (Figure 12c). These hybrid nanoparticles preserved the functional proteins on the EV surface and effectively delivered COL1A1 mRNA to stem cells [151]. Similarly, Cardellini et al. used a microfluidic ultrasonic device (μSonicator) to effectively coat poly (methyl methacrylate) (PMMA) nanoparticles with liposomes and EVs, achieving a high coating efficiency of up to 70% while maintaining particle uniformity and consistency of the coating [32]. Collectively, these studies highlight the critical role of microfluidic technologies in fabricating EV–nanoparticle hybrid systems, underscoring their potential to advance targeted drug delivery and therapeutic strategies.

## 4. Integrated Microfluidic Strategies: From Intracellular Engineering to Therapeutic Carrier Development

As discussed earlier, microfluidic technology has demonstrated significant potential not only in the precise intracellular delivery of nucleic acids [28], but also in the fabrication and engineering of effective therapeutic carriers [30]. Current research trends are progressing beyond employing these two strategies separately, instead moving toward integrated strategies. These include directly delivering nucleic acids into cells to generate EVs endowed with therapeutic activity [152,153], or coupling engineered cells with hydrogels for direct tissue and organ delivery [154]. Such integrated strategies synergistically combine the strengths of each individual method, enhancing therapeutic efficacy and suggesting novel application possibilities that address the limitations of conventional approaches.

### 4.1. Engineering of Cells for EV-Mediated Gene Therapy

**Figure 13:**
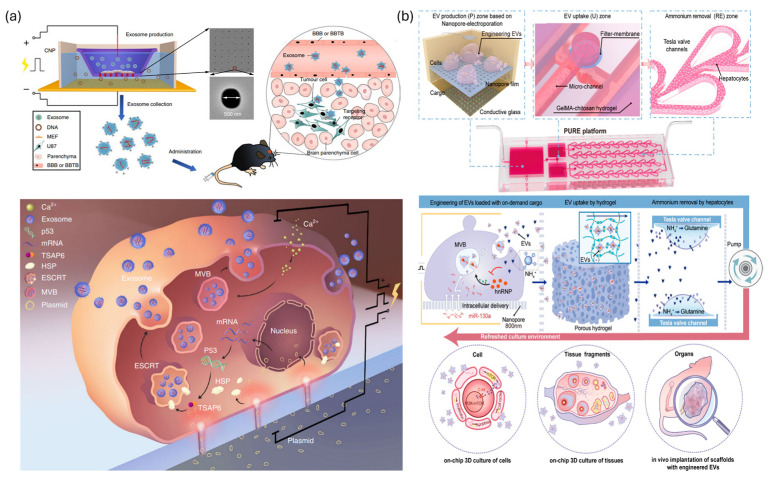
Integrated microfluidic platforms for therapeutic EV engineering. (**a**) A schematic of the Cellular Nanoporation (CNP) platform using nanostructured electrodes to stimulate exosome release with high mRNA loading efficiency. Reprinted and modified from Ref. [152]. (**b**) A schematic illustration of the PURE platform integrating electroporation, EV harvesting, and waste removal within a single device to enable continuous, high-yield production of therapeutic EVs. Reprinted and modified from Ref. [153].

Despite earlier microfluidic advances in EV production, a key limitation remained: conventional methods produced EVs carrying only the cell’s own biomolecular content [84,143,144,145]. This meant that any desired therapeutic nucleic acids had to be loaded into the vesicles as a separate post-production step [146]. Recent integrated strategies address this shortcoming by combining intracellular delivery with EV secretion in a single process. In these integrated systems, specific nucleic acids or other therapeutic agents are delivered directly into cells simultaneously with EV release stimulation, yielding vesicles already loaded with the introduced cargo. By unifying cargo loading and EV generation into one streamlined operation, such approaches eliminate the need for multi-step processing and mark a significant step toward fully self-contained EV-based therapeutic carrier platforms [152,153]. A prime example is the cellular nanoporation (CNP) platform, which employs localized, transient electrical pulses via nano-channels to stimulate cells to release exosomes loaded with therapeutic mRNA (Figure 13a). Recent studies demonstrated that CNP biochips achieved exosome production rates up to 50-fold greater compared to conventional bulk electroporation, and increased mRNA loading by over 1000-fold. Notably, even cells with inherently low EV secretion capabilities successfully generated substantial amounts of EVs containing therapeutic mRNA using this approach. These engineered EVs demonstrated biological functionality; for instance, delivering tumor suppressor genes effectively inhibited tumor growth in a brain tumor model [152].

Another integrated strategy is the Production–Uptake–Removal (PURE) microfluidic platform, which concurrently engineers and collects EVs within a single integrated device. Individual cells are locally electroporated through nanopore membranes in the “production zone,” which performs intracellular delivery of therapeutic nucleic acids (e.g., miR-130a mimics) and stimulates EV secretion (Figure 13b). Generated EVs are immediately captured in an adjacent hydrogel-based “collection zone,” achieving capture efficiencies exceeding 85%. Additionally, the platform includes a downstream toxicant removal zone containing hepatocytes to clear cellular waste products, maintaining cell viability and supporting sustained high-quality EV production. The PURE platform demonstrated its capability by significantly increasing EV yield approximately 10-fold over natural secretion and enhancing miRNA concentration more than 100-fold. In a mouse model, EV-loaded hydrogels directly implanted into aging ovaries showed therapeutic efficacy by restoring ovarian function [153]. These results highlight the potential of integrating ex vivo EV engineering technologies with biocompatible delivery vehicles, positioning platforms such as CNP and PURE as promising candidates for next-generation optimized therapeutic carrier systems.

### 4.2. Hydrogel-Integrated Stem Cell Systems for Targeted Therapeutic Release

**Figure 14:**
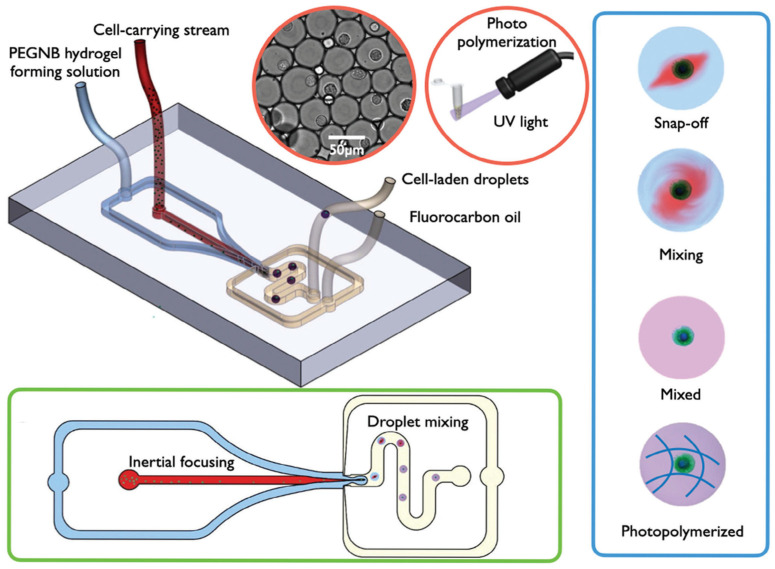
A schematic of a microfluidic platform generating uniform PEG-based microgels for single MSC encapsulation, enhancing cell survival and sustained anti-inflammatory cytokine release in vivo. Reprinted and modified from Ref. [154].

Concurrently, considerable research efforts have focused on developing biocompatible hydrogels to facilitate direct cell delivery into tissues and organs, aiming to maintain cellular viability and functional persistence. A recent study introduced a novel approach to encapsulate mesenchymal stem cells (MSCs) within polyethylene glycol (PEG)-based hydrogels to improve their survival and sustained functionality post-transplantation (Figure 14). Uniformly sized PEG hydrogels (~100 µm) were generated via microfluidics, individually encapsulating MSCs. The encapsulated MSCs were effectively protected by the hydrogel environment, enhancing their survival in the hostile in vivo conditions and significantly improving their therapeutic efficacy compared to free MSCs.

Specifically, when applied in a mouse inflammation model, the encapsulated MSCs exhibited prolonged secretion of anti-inflammatory cytokines, including IL-10 and TGF-β, thereby reducing tissue damage and demonstrating superior therapeutic outcomes [154]. These findings highlight the potential of hydrogel-based cell encapsulation platforms to provide sustained therapeutic signaling within the body. Currently, this delivery platform does not include intracellular nucleic acid delivery; however, integration with microfluidics-based intracellular delivery technologies is expected to enhance their therapeutic capabilities [25]. For example, microfluidic cell nanoporation could be utilized to pre-load mRNA or gene editing factors into MSCs [155], followed immediately by encapsulation in PEG microgels [156] or microneedles [157] for direct implantation. Such an approach could facilitate the development of more sophisticated cell-based therapies. The integration of intracellular nucleic acid delivery with targeted delivery strategies for tissues and organs represents a promising convergence strategy, offering significant potential to further enhance the precision and therapeutic efficacy of cell-based regenerative medicine.

## 5. Conclusions and Perspectives

Recent clinical successes with NATs have opened new horizons in precision therapies for genetic disorders, cancer, infectious diseases, and beyond. However, the full realization of these breakthroughs requires overcoming critical technological limitations—commonly referred to as the “delivery problem”—which involve accurately and safely delivering therapeutic agents to specific target cells. Microfluidic platforms have emerged as a versatile solution to address these challenges, providing a unified technological framework applicable to both intracellular delivery and therapeutic carrier development. Microfluidic platforms offer considerable potential to enhance nucleic acid delivery efficiency and stability throughout the entire therapeutic process—from cellular-level nucleic acid introduction to the manufacturing of carriers for targeted tissue and organ delivery. Various microfluidic platforms, including mechanical permeation via cell deformation, microfluidic electroporation, and microfluidic sonoporation, have been developed to efficiently deliver nucleic acids into cells while minimizing cell damage and maximizing cell viability. Meanwhile, microfluidic-based nanoparticle manufacturing technology has played a key role in the success of mRNA vaccines and siRNA therapeutics by enabling the production of delivery vehicles such as lipid nanoparticles (LNPs), polymeric nanoparticles, and extracellular vesicles (EVs) with superior uniformity and reproducibility compared to conventional bulk processes. In particular, microfluidic processes have significantly contributed to enhancing in vivo stability and target cell specificity by precisely controlling particle size and surface characteristics. Furthermore, integrated strategies combining multiple delivery technologies have become achievable through microfluidic platforms. Novel approaches that bridge the boundaries between cell therapy and therapeutic carrier engineering are actively being pursued. Examples include simultaneous intracellular delivery and EV secretion induction on a single microfluidic platform, or ex vivo encapsulation of genetically engineered cells into hydrogels via microfluidics for subsequent direct administration into patients. These integrated strategies promise to amplify synergies throughout the NATs process, enabling simultaneous genetic manipulation at the cellular level and precise drug delivery at the tissue and organ levels within a unified platform.

Microfluidic-based NATs platforms are expected to advance further in several key aspects in the future. First, successful ex vivo gene therapy manufacturing requires integrating sensors into microfluidic systems to enable real-time quality control. These devices can then continuously monitor important parameters, such as cell viability, transfection efficiency, and nucleic acid uptake, immediately after delivery. This real-time monitoring enables the quick identification of successfully modified cells and helps ensure the consistency and safety of the final product. However, since expressing and validating nucleic acid function often takes several hours to days, a fully integrated platform should include on-chip cell culture modules for post-delivery incubation and monitoring. Such comprehensive systems, combining delivery, sensing [158], and cell culture [159], would enable automated, high-throughput, quality-assured gene therapy manufacturing, representing a significant advancement for the field. Second, while the optimization of manufacturing parameters such as FRR and TFR using machine learning algorithms (e.g., Bayesian optimization [160], XGBoost models [161]) has been successfully demonstrated, the future direction lies in integrating these optimization frameworks with real-time adaptive control systems. This integration will enable continuous, automated adjustments during manufacturing, significantly enhancing reproducibility and quality control. Additionally, advanced machine learning techniques capable of efficiently navigating higher-dimensional and multi-objective parameter spaces will be critical for simultaneously optimizing complex therapeutic requirements, such as targeted cellular uptake, minimal cytotoxicity, and maximal nucleic acid delivery efficiency. Third, the convergence of nanomaterial technologies with biomaterials such as hydrogels and nanogels presents substantial potential for enhancing the long-term stability of therapeutic carriers within in vivo environments. This synergy also facilitates precise control over therapeutic agent release profiles. For instance, stimuli-responsive nanogels have demonstrated the controlled modulation of mRNA release rates in response to environmental cues such as pH or temperature [162]. Additionally, hyaluronan-based hydrogels have shown the capability to sustain gene expression within target tissues for periods exceeding 30 days [163]. Fourth, integrating precision medicine driven by genomic analysis with patient-specific NAT development makes personalized gene therapy increasingly feasible. Combining AI-based interpretive algorithms [164] with microfluidic platforms for single-cell genomic analysis could enable the real-time optimization of LNP compositions tailored to individual patients’ cellular characteristics. Furthermore, the development of customized EV production platforms for CRISPR-Cas9 delivery holds transformative potential for advancing personalized gene-editing therapies [165].

In summary, microfluidic platforms represent a robust solution to the longstanding “delivery problem,” a critical hurdle impeding the widespread development of NATs. With advantages including high delivery efficiency, excellent cell viability, and high-throughput processing capabilities, microfluidic platforms offer significant promise as integrated solutions poised to play a central role in the future of precision gene and cell therapies.

## Supplementary Materials

The following supporting information can be downloaded at https://www.mdpi.com/article/10.3390/bios15080504/s1, Table S1: FDA-approved NATs based on ex vivo gene therapy; Table S2: FDA-approved NATs based on in vivo gene therapy; Table S3: Summary of Microfluidic Platforms for Intracellular Nucleic Acid Delivery.
