# Supplementary material for: Microfluidic Platforms for Ex Vivo and In Vivo Gene Therapy
Source: Biosensors (Basel). 2025 Aug 4;15(8):504. doi: 10.3390/bios15080504 (PMC12384408; doi:10.3390/bios15080504)
Supplement: Supplementary file 1 [file biosensors-15-00504-s001.zip › biosensors-3705266-supplementary.pdf]

biosensors MDPI

Supplementary Materials 1

Table S1. FDA-approved NATs based on *Ex vivo* gene therapy 2

Type	Drug (Brand name)	Company	Indication	Target (organ)	Mechanism	FDA Approval	Ref(s)
CAR-T	Kymriah (tisagenlecleucel)	Novartis	r/r B-ALL (≤25 yrs)	Hematologic (B-cells)	Lentiviral CAR gene insertion (CD19 targeting)	2017	[1]
	Yescarta (axicabtagene ciloleucel)	Kite Pharma (Gilead)	r/r LBCL	Hematologic (B-cells)	Gamma-retroviral CAR gene insertion (CD19 targeting)	2017	[2]
	Tecartus (brexucabtagene autoleucel)	Kite Pharma (Gilead)	MCL / aALL	Hematologic (B-cells)	Gamma-retroviral CAR gene insertion (CD19 targeting)	2020	[3]
	Breyanzi (lisocabtagene maraleucel)	Juno Therapeutics (BMS)	r/r LBCL	Hematologic (B-cells)	Lentiviral CAR gene insertion (CD19 targeting)	2021	[4]
	Abecma (idecabtagene vicleucel)	Bristol Myers Squibb / bluebird bio	r/r MM	Hematologic (plasma cells)	Lentiviral CAR gene insertion (BCMA targeting)	2021	[5]
	Carvykti (ciltacabtagene autoleucel)	Janssen / Legend Biotech	r/r MM	Hematologic (plasma cells)	Lentiviral CAR gene insertion (BCMA targeting)	2022	[6]
	Aucatzyl (obecabtagene autoleucel)	Autolus Therapeutics	r/r adult B-ALL	Hematologic (B-cells)	Lentiviral CAR gene insertion (CD19 targeting)	2024	[7]
TCR-T (MAGE-A4)	Tecelra (afamitresgene autoleucel)	Adaptimmune Therapeutics	Adv. SS (HLA-A2+, MAGE-A4+)	Solid tumor	Lentiviral TCR gene insertion (MAGE-A4 targeting)	2024	[8]
HSC gene therapy	Zynteglo (betibeglogene autotemcel)	bluebird bio	TDT β-thal	Bone marrow	Lentiviral addition of β-globin gene	2022	[9]
	Skysona (elivaldogene autotemcel)	bluebird bio	cALD (boys 4–17 yrs)	CNS (oligodendrocytes)	Lentiviral addition of ABCD1 gene	2022	[10]
	Lenmeldy (atidarsagene autotemcel)	Orchard Therapeutics	E-MLD	CNS (oligodendrocytes)	Lentiviral addition of ARSA gene	2024	[11]

	Lyfgenia (lovotibeglogene autotemcel)	bluebird bio	SCD (≥12 yrs)	Bone marrow	Lentiviral addition of T87Q β-globin gene	2023	[12]
CRISPR-Cas9	Casgevy (exagamglogene autotemcel)	Vertex / CRISPR Therapeutics	SCD / β-thal	Bone marrow	CRISPR-Cas9 disruption of BCL11A gene	2023	[13]

Table S2. FDA-approved NATs based on *in vivo* gene therapy

Type	Drug (Brand name)	Company	Indication	Target (organ)	Mechanism	FDA Approval	Ref(s)
ASO	Eteplirsen (Exondys 51)	Sarepta Therapeutics	DMD	Exon 51 of DMD (muscle)	Induces skipping of exon 51 in dystrophin pre-mRNA to produce a partially functional dystrophin protein	2016	[14, 15]
	Nusinersen (Spinraza)	Ionis & Biogen	SMA	Exon 7 of SMN2 (CNS)	Modulates splicing of the SMN2 gene to increase the production of functional SMN protein	2016	[16, 17]
	Inotersen (Tegsedi)	Ionis	hATTR-PN	TTR (liver)	Degrades TTR mRNA in hepatocytes to lower serum TTR protein levels and prevent amyloid accumulation	2018	[18, 19]
	Golodirsen (Vyondy 53)	Sarepta Therapeutics	DMD	Exon 53 of DMD (muscle)	Induces skipping of exon 53 in dystrophin pre-mRNA to promote the production of functional dystrophin	2019	[20]
	Viltolarsen (Viltepso)	Nippon Shinyaku	DMD	Exon 53 of DMD (muscle)	Induces exon 53 skipping to produce truncated but functional dystrophin protein	2020	[21, 22]
	Casimersen (Amondys 45)	Sarepta Therapeutics	DMD	Exon 45 of DMD (muscle)	Induces skipping of exon 45 in dystrophin pre-mRNA to enable the production of functional dystrophin protein	2021	[23, 24]
	Tofersen (Qalsody)	Biogen	SOD1-ALS	SOD1(CNS)	Degrades SOD1 mRNA to suppress the expression of toxic SOD1 protein and slow neuronal damage	2023	[25, 26]

TCR-T (MAGE-A4)	Eplontersen (Wainua)	Ionis & AstraZeneca	ATTRv-PN	TTR (liver)	Selectively degrades TTR mRNA in hepatocytes to reduce serum TTR levels and prevent amyloid formation	2023	[27]
siRNA	Patisiran (Onpattro)	Alnylam	hATTR-PN	TTR (liver)	Degrades TTR mRNA via the siRNA-RISC complex in hepatocytes to inhibit TTR protein synthesis	2018	[28]
	Givosiran (Givlaari)	Alnylam	AHP	ALAS1(liver)	Targets ALAS1 mRNA for degradation in hepatocytes to reduce the production of toxic porphyrins	2019	[29]
	Lumasiran (Oxlumo)	Alnylam	PH1	HAO1(liver)	Degrades HAO1 mRNA in hepatocytes to reduce the production of oxalate	2020	[30]
	Inclisiran (Leqvio)	Alnylam & Novaetis	primary hypercholesterolemia	PCSK9(liver)	Targets PCSK9 mRNA in hepatocytes for degradation to lower LDL cholesterol levels	2021	[31]
CRISPR-Cas9	Vutrisiran (Amvuttra)	Alnylam	hATTR-PN	TTR (liver)	Targets TTR mRNA for degradation in hepatocytes to reduce TTR protein levels and amyloid accumulation	2022	[32]

Table S3. Summary of Microfluidic Platforms for Intracellular Nucleic Acid Delivery

Delivery Method	Platform(Technique)	Cell Line	Nucleic Acid Type	Concentration	Transfection Efficiency	Viability	Throughput	Ref.
Cell deformation	Elastic Deformability Microfluidics	HEK293 FT	pDNA(GFP)	0.8 μg/mL	~42%	>80%	$1.2 \times 10^4\ cells/min$	[33]
	Flexible pneumatic microvalve array	MCF-7	mRNA (EGFP)<br>pDNA (pCMV-LifeAct RFP)	10 μg/mL<br>N/A	90%<br>95.5%	>85%	$2.0 \times 10^6\ cells/min$	[34]
	Droplet Cell Pincher	K562	mRNA<br>pDNA<br>Cas9 RNP	2ug/ml<br>50ug/ml<br>100ug/ml	99%<br>91%<br>50% (knockout)	~75 %	$1 \times 10^5\ cells/min$	[35]
	Y-Hydroporator	Human primary NK cells	mRNA<br>Cas9 RNP:	50 μg mL^{-1}<br>2ug/ml	~74% (CAR expression)<br>~37% (gene editing)<br>~18.56% (KO efficiency)	>89%	$2 \times 10^6\ cells/min$	[36]
	Microfluidic Vortex Shedding	Primary human T cells ($CD3^+$)	Cas9 RNP	Cas9 ~40 μM	40%(knockout efficiency)	60–70%	$2.5 \times 10^8\ cells/min$	[37]
	Hydroporation (viscoelastic)	Jurkat	mRNA<br>Cas9 RNP	100 μg mL^{-1}<br>1 μM	~89%<br>~53% TCR knockout	74%<br>66–73%	$2.5 \times 10^8\ cells/min$	[38]
Microfluidic electroporation	Nanopore Electroporation Device (NPE)	HeLa<br>DC2.4<br>HL-1<br>Raw264.7<br>Jurkat	pDNA(GFP)	100μg/ml	58%<br>68%<br>15.8%<br>4%<br>2%	93%<br>90%<br>90%<br>97%<br>100%	$8 \times 10^3\ cells/device$<br>$8 \times 10^3\ cells/device$<br>$5 \times 10^3\ cells/device$<br>$1.5 \times 10^4\ cells/device$<br>$3 \times 10^4\ cells/device$	[39]
	Nanoneedle Array Electroporation	Primary BMDCs	Cas9 RNP	0.5 μM	40% (PD-L1 knockout)	>96%	$3 \times 10^4\ cells/device$	[40]

 https://doi.org/10.3390/xxxxx

	Continuous-flow Electroporation	Primary human t cells	mRNA<br>pDNA<br>Cas9 RNP	50µg/ml<br>100µg/ml<br>4 µM	93%<br>86%<br>93%	99%<br>70%<br>99%	$1.6 \times 10^8\ cells/min$<br>$4 \times 10^6\ cells/min$<br>$1.6 \times 10^8\ cells/min$	[41]
	Multiplexed Continuous-flow Electroporation	Jurkat T	pDNA(GFP)	37.5 µg/mL	~85%	~95%	$8 \times 10^6\ cells/min$	[42]
	LaViE-Chip	HEK293T	pDNA	100µg/ml	71.06%	84.3%	$1 \times 10^7\ cells/min$	[43]
Microfluidic sonoporation	Acoustofluidic sonoporation	Jurkat<br>PBMCs<br>HSPCs	pDNA	100µg/ml	62%<br>15%<br>20%	80%<br>92%<br>80%	$1.95 \times 10^5\ cells/min$<br>$1.95 \times 10^5\ cells/min$<br>$3.25 \times 10^4\ cells/min$	[44]
	Acoustothermal Transfection	MCF-7<br>MSC<br>Primary T	pDNA	10 ng/ul	96%<br>89%<br>80%	85.9%<br>83.9%<br>84.4%	$1.7\ 10^6\ cells/min$	[45]
	Acoustic-Electric Shear Orbiting Poration (AESOP)	HeLa<br>K562<br>Jurkat	pDNA (eGFP, CRISPR-Cas9)	N/A	>80% (6.1 kbp eGFP)<br>~20% KO (9.3 kbp CRISPR)<br>>40% (6.1 kbp eGFP)	80%	$1 \times 10^6\ cells/min$	[46]

References

1. Maude, S.L., et al., *Tisagenlecleucel in children and young adults with B-cell lymphoblastic leukemia.* New England Journal of Medicine, 2018. **378**(5): p. 439-448.
2. Neelapu, S.S., et al., *Axicabtagene ciloleucel CAR T-cell therapy in refractory large B-cell lymphoma.* New England Journal of Medicine, 2017. **377**(26): p. 2531-2544.
3. Wang, M., et al., *KTE-X19 CAR T-cell therapy in relapsed or refractory mantle-cell lymphoma.* New England journal of medicine, 2020. **382**(14): p. 1331-1342.
4. Abramson, J.S., et al., *Lisocabtagene maraleucel for patients with relapsed or refractory large B-cell lymphomas (TRANSCEND NHL 001): a multicentre seamless design study.* The Lancet, 2020. **396**(10254): p. 839-852.
5. Munshi, N.C., et al., *Idecabtagene vicleucel in relapsed and refractory multiple myeloma.* New England Journal of Medicine, 2021. **384**(8): p. 705-716.
6. Berdeja, J.G., et al., *Ciltacabtagene autoleucel, a B-cell maturation antigen-directed chimeric antigen receptor T-cell therapy in patients with relapsed or refractory multiple myeloma (CARTITUDE-1): a phase 1b/2 open-label study.* The Lancet, 2021. **398**(10297): p. 314-324.
7. Roddie, C., et al., *Obecabtagene autoleucel in adults with B-cell acute lymphoblastic leukemia.* New England Journal of Medicine (NEJM), 2024.
8. D'Angelo, S.P., et al., *Afamitresgene autoleucel for advanced synovial sarcoma and myxoid round cell liposarcoma (SPEARHEAD-1): an international, open-label, phase 2 trial.* The Lancet, 2024. **403**(10435): p. 1460-1471.
9. Locatelli, F., et al., *Betibeglogene autotemcel gene therapy for non–β0/β0 genotype β-thalassemia.* New England Journal of Medicine, 2022. **386**(5): p. 415-427.
10. Eichler, F., et al., *Hematopoietic stem-cell gene therapy for cerebral adrenoleukodystrophy.* New England Journal of Medicine, 2017. **377**(17): p. 1630-1638.
11. Fumagalli, F., et al., *Lentiviral haematopoietic stem-cell gene therapy for early-onset metachromatic leukodystrophy: long-term results from a non-randomised, open-label, phase 1/2 trial and expanded access.* The Lancet, 2022. **399**(10322): p. 372-383.
12. Kanter, J., et al., *Biologic and clinical efficacy of LentiGlobin for sickle cell disease.* New England Journal of Medicine, 2022. **386**(7): p. 617-628.
13. Philippidis, A., *CASGEVY makes history as FDA approves first CRISPR/Cas9 genome edited therapy.* Human gene therapy, 2024. **35**(1-2): p. 1-4.
14. Syed, Y.Y., *Eteplirsen: first global approval.* Drugs, 2016. **76**: p. 1699-1704.
15. Cirak, S., et al., *Exon skipping and dystrophin restoration in patients with Duchenne muscular dystrophy after systemic phosphorodiamidate morpholino oligomer treatment: an open-label, phase 2, dose-escalation study.* The Lancet, 2011. **378**(9791): p. 595-605.
16. Finkel, R.S., et al., *Nusinersen versus sham control in infantile-onset spinal muscular atrophy.* New England Journal of Medicine, 2017. **377**(18): p. 1723-1732.
17. Hoy, S.M., *Nusinersen: first global approval.* Drugs, 2017. **77**(4): p. 473-479.
18. Benson, M.D., et al., *Inotersen treatment for patients with hereditary transthyretin amyloidosis.* New England Journal of Medicine, 2018. **379**(1): p. 22-31.
19. Keam, S.J., *Inotersen: first global approval.* Drugs, 2018. **78**: p. 1371-1376.
20. Heo, Y.-A., *Golodirsen: first approval.* Drugs, 2020. **80**(3): p. 329-333.
21. Clemens, P.R., et al., *Safety, tolerability, and efficacy of viltolarsen in boys with Duchenne muscular dystrophy amenable to exon 53 skipping: a phase 2 randomized clinical trial.* JAMA neurology, 2020. **77**(8): p. 982-991.
22. Dhillon, S., *Viltolarsen: first approval.* Drugs, 2020. **80**(10): p. 1027-1031.

23. Shirley, M., *Casimersen: first approval.* Drugs, 2021. **81**: p. 875-879.
24. Wagner, K.R., et al., *Safety, tolerability, and pharmacokinetics of casimersen in patients with D uchenne muscular dystrophy amenable to exon 45 skipping: A randomized, double-blind, placebo-controlled, dose-titration trial.* Muscle & nerve, 2021. **64**(3): p. 285-292.
25. Blair, H.A., *Tofersen: first approval.* Drugs, 2023. **83**(11): p. 1039-1043.
26. Miller, T.M., et al., *Trial of antisense oligonucleotide tofersen for SOD1 ALS.* New England Journal of Medicine, 2022. **387**(12): p. 1099-1110.
27. Coelho, T., et al., *Eplontersen for hereditary transthyretin amyloidosis with polyneuropathy.* Jama, 2023. **330**(15): p. 1448-1458.
28. Hoy, S.M., *Patisiran: first global approval.* Drugs, 2018. **78**: p. 1625-1631.
29. Syed, Y.Y., *Givosiran: a review in acute hepatic porphyria.* Drugs, 2021. **81**(7): p. 841-848.
30. Scott, L.J. and S.J. Keam, *Lumasiran: first approval.* Drugs, 2021. **81**: p. 277-282.
31. Lamb, Y.N., *Inclisiran: first approval.* Drugs, 2021. **81**: p. 389-395.
32. Keam, S.J., *Vutrisiran: first approval.* Drugs, 2022. **82**(13): p. 1419-1425.
33. Alhmoud, H., et al., *Leveraging the elastic deformability of polydimethylsiloxane microfluidic channels for efficient intracellular delivery.* Lab on a Chip, 2023. **23**(4): p. 714-726.
34. Qu, J., et al., *Flexible Mechanoporation Chips for High-Throughput Intracellular Delivery Based on Controlled Pneumatic Microvalve Array.* ACS Nano, 2025. **19**(24): p. 22017-22031.
35. Kim, Y.-J., et al., *Highly efficient CRISPR-mediated genome editing through microfluidic droplet cell mechanoporation.* Nature Communications, 2024. **15**(1).
36. Kim, H., et al., *Advancing Allogeneic NK Cell Immunotherapy through Microfluidic Gene Delivery.* Advanced Science, 2025.
37. Sytsma, B.J., et al., *Scalable intracellular delivery via microfluidic vortex shedding enhances the function of chimeric antigen receptor T-cells.* Scientific Reports, 2025. **15**(1).
38. Sevenler, D. and M. Toner, *High throughput intracellular delivery by viscoelastic mechanoporation.* Nature Communications, 2024. **15**(1).
39. Liu, J., et al., *Nanopore Electroporation Device for DNA Transfection into Various Spreading and Nonadherent Cell Types.* ACS Applied Materials & Interfaces, 2023. **15**(43): p. 50015-50033.
40. Liu, X., et al., *Nanoneedle Array-Electroporation Facilitates Intranuclear Ribonucleoprotein Delivery and High Throughput Gene Editing.* Advanced Healthcare Materials, 2024. **13**(29).
41. Welch, M., et al., *High-Throughput CRISPR/Cas9 Mediated Gene Editing of Primary Human T Cells in a Microfluidic Device for Cellular Therapy Manufacturing.* Advanced Materials Technologies, 2023. **8**(17).
42. Vanderburgh, J.A., et al., *A multiplexed microfluidic continuous-flow electroporation system for efficient cell transfection.* Biomedical Microdevices, 2024. **26**(2).
43. Li, Z., et al., *Expanding the cell quantity of CRISPR/Cas9 gene editing by continuous microfluidic electroporation chip.* Bioelectrochemistry., 2025. **161**: p. 108840.
44. Belling, J.N., et al., *Acoustofluidic sonoporation for gene delivery to human hematopoietic stem and progenitor cells.* Proceedings of the National Academy of Sciences, 2020. **117**(20): p. 10976-10982.
45. Liu, X., et al., *Acoustothermal transfection for cell therapy.* Science Advances, 2024. **10**(16): p. eadk1855.
46. Aghaamoo, M., et al., *High-Throughput and Dosage-Controlled Intracellular Delivery of Large Cargos by an Acoustic-Electric Micro-Vortices Platform.* Advanced Science, 2022. **9**(1): p. 2102021.
